# Optimizing the fermentation parameters in the Lactic Acid Fermentation of Legume-based Beverages– a statistically based fermentation

**DOI:** 10.1186/s12934-024-02522-x

**Published:** 2024-09-19

**Authors:** Stefan W. Ritter, Quentin P. Thiel, Martina I. Gastl, Thomas M. Becker

**Affiliations:** 1 Institute of Brewing and Beverage Technology, Technical University Munich, 85354 Freising, Germany; 2 Research Center Weihenstephan for Brewing and Food Quality, Technical University Munich, 85354 Freising, Germany

**Keywords:** Lactic acid bacteria, Faba beans, Lupines, Design of experiment, Process optimization, Refreshing beverage, Beany aroma impression, Protein-rich beverage

## Abstract

**Background:**

The market for beverages is highly changing within the last years. Increasing consumer awareness towards healthier drinks led to the revival of traditional and the creation of innovative beverages. Various protein-rich legumes were used for milk analogues, which might be also valuable raw materials for refreshing, protein-rich beverages. However, no such applications have been marketed so far, which might be due to unpleasant organoleptic impressions like the legume-typical “beany” aroma. Lactic acid fermentation has already been proven to be a remedy to overcome this hindrance in consumer acceptance.

**Results:**

In this study, a statistically based approach was used to elucidate the impact of the fermentation parameters temperature, inoculum cell concentration, and methionine addition on the fermentation of lupine- and faba bean-based substrates. A total of 39 models were found and verified. The majority of these models indicate a strong impact of the temperature on the reduction of aldehydes connected to the “beany” impression (e.g., hexanal) and on the production of pleasantly perceived aroma compounds (e.g., β-damascenone). Positively, the addition of methionine had only minor impacts on the negatively associated sulfuric compounds methional, dimethyl sulfide, dimethyl disulfide, and dimethyl trisulfide. Moreover, in further fermentations, the time was added as an additional parameter. It was shown that the strains grew well, strongly acidified the both substrates (pH ≤ 4.0) within 6.5 h, and reached cell counts of > 9 log_10_ CFU/mL after 24 h. Notably, most of the aldehydes (like hexanal) were reduced within the first 6–7 h, whereas pleasant compounds like β-damascenone reached high concentrations especially in the later fermentation (approx. 24–48 h).

**Conclusions:**

Out of the fermentation parameters temperature, inoculum cell concentration, and methionine addition, the temperature had the highest influence on the observed aroma and taste active compounds. As the addition of methionine to compensate for the legume-typical deficit did not lead to an adverse effect, fortifying legume-based substrates with methionine should be considered to improve the bioavailability of the legume protein. Aldehydes, which are associated with the “beany” aroma impression, can be removed efficiently in fermentation. However, terminating the process prematurely would lead to an incomplete production of pleasant aroma compounds.

**Supplementary Information:**

The online version contains supplementary material available at 10.1186/s12934-024-02522-x.

## Background

Fermentation has been used for millennia in the production and preservation of food and beverages. Examples of very traditional products like beer, bread, or vinegar, are present all around the globe. However, especially in the beverage sector, innovative product applications or the revival of ancient products in new markets are constantly gaining attention. Such innovations could be using new yeast strains for the production of low-alcohol beers [1, 2], the rise of the traditional South East Asian fermented tea kombucha in Europe and the US [3], the adaption of the brewing process to produce gluconic acid fermented refreshing lemonades [1], or the fermentation of fruit juices to reduce the sugar content, to enhance the shelf life or to add a probiotic character [4]. This increasing demand for healthy refreshing beverages goes along with a rising consumer awareness towards nutritional aspects [5]. Especially legumes are particularly suitable for the production of functional protein-rich beverages, as they contain high amounts of vegan protein (up to 50% [6, 7]). However, while there are plenty of milk alternatives based on soy, lupine, or pea commercially available [8], no legume-based refreshing beverages are available, so far.

This missing product introduction can be explained with several hurdles which need to be overcome first. Their major disadvantages are the low protein solubility in the acidic pH range of refreshing beverages [9–13] and their distinctive “green” and “beany” aroma impression [8]. While the first disadvantage can be mitigated by germination [9] and enzymatic treatment [10, 14], the second issue can be resolved by lactic acid fermentation [15–18]. Besides the aroma improvement, the fermentation with lactic acid bacteria (LAB) introduces pleasant organic acids (e.g., lactic acid), which add to the refreshing character of the beverage.

The result of a LAB fermentation depends highly on the selected strain and on the fermentation parameters. High variations in the resulting aroma spectrum were studied in soy [19], lupine, and faba bean [20]. The high impact of the fermentation temperature and the inoculum cell concentration on the resulting aroma profile or sensory attributes were described for the fermentation of beer [21] and kwass [22]. A further concern when using legumes is their lack of sulfuric amino acids (e.g., methionine) [7, 23, 24]. This might affect the fermentation itself as lactic acid bacteria have limited abilities to synthesize amino acids and, therefore, require a well-balanced amino acid spectrum in the substrate (as reviewed by Savijoki, Ingmer [25]). Furthermore, a methionine deficit leads directly to a reduced bioavailability of the overall protein. Therefore, the addition of methionine to a legume-based substrate seems to be a solution to mitigate both disadvantages.

However, via metabolic pathways present in LAB [26], methionine is directly connected to the aldehyde methional (aroma impression described as “potato-like” [27]) and via homocysteine to the sulfuric aroma compounds dimethyl sulfide (DMS) (“sulfuric, canned corn” [27]), dimethyl disulfide (DMDS) (“sulfuric, cabbage-like” [27]), and dimethyl trisulfide (DMTS) (“sulfuric, cabbage-like, onion-like” [27]). As all these aroma compounds are described with unpleasant aroma impressions, the addition of methionine needs to be evaluated carefully.

In this study, the impact of the fermentation parameters temperature, inoculum cell concentration, and methionine addition on the lactic acid fermentation of lupine and faba bean-based substrates was evaluated. To identify and evaluate not only the impact of the three fermentation parameters isolated but also of interacting effects, a statistical design of experiment was applied instead of one-factor-at-a-time experiments.

A variety of aroma- and taste-active compounds were chosen as response variables to build models and evaluate the impact of the fermentation parameters. Those were the sulfuric aroma compounds mentioned above: methional, DMS, DMDS, and DMTS. As legumes contain mainly unsaturated and polyunsaturated fatty acids (like linoleic acid or α-linolenic acid [15]), oxidation products like hexanal, (*E*)-non-2-enal, or (2*E*,4*E*)-nona-2,4-dienal are intensely involved in forming the distinctive “beany” aroma of legumes [28, 29]. (*E*)-non-2-enal does not only participate in forming this “beany” odor [29], it is also known for its distinctive “cardboard-like” off-flavor in beer [30]. Therefore, the concentration of those aldehydes should be minimized upon fermentation. Hexanal can be reduced to hexanol or oxidized to hexanoic acid. Subsequently, the latter can react with ethanol to ethyl hexanoate (“fruity” [27]), which would improve the aroma acceptance in legume-based refreshing beverages. Linoleic acid is also the precursor to the pleasant fermentation product γ-nonalactone (“coconut,” “fruity” [27]), which would also be very appropriate in a refreshing beverage. The diketone diacetyl (“buttery,” “sweet” [27]) is known to contribute very positively to the aroma of yogurt [31], is often associated with LAB, and might therefore contribute positively if the concentration does not exceed a moderate level. Diacetyl evolves from pyruvate by pyruvate dehydrogenase or acetolactate synthase via α-acetolactate and subsequent non-enzymatic oxidation [32] or enzymatic oxidation of acetoin [32]. For faba beans, it was shown that the carotenoid-derived β-damascenone (“cooked apple,” “honey-like,” “fruity” [27]) increases very strongly in the malting process (steeping, germination and drying) [33]. As this aroma compound would be very pleasant for a refreshing beverage, changes in its concentration were monitored in the LAB fermentation, too. For the taste perception, a balanced acidity is required. Homofermentative LAB produce mainly lactic acid via the Embden-Meyerhof-Parnas pathway [32]. However, if oxygen or electron acceptors are available in the fermentation, acetic acid can be formed from pyruvate via acetyl-phosphate [32]. As the taste of acetic acid is described as “pungent” and negatively perceived [34–36], the production of lactic acid is preferable.

This study can be divided into three consecutive steps. After identifying models to predict the results of the fermentations (step 1), those were validated in separate experiments with fermentation parameters chosen within the design space but different from the initial ones (step 2). To gain a deeper understanding of the time-dependent changes during the fermentation and to evaluate changes in the metabolic activities, further fermentations with both legumes were performed in the highly controlled environment of a bioreactor (step 3).

## Materials and methods

### Chemicals

All calibrations and identifications using gas and liquid chromatography were performed with commercially available analytical standard compounds. The following aroma standards were purchased from Sigma-Aldrich (Steinheim, Germany): 3-methylbutanal (CAS 590-86-3), 2-methylbutanal (CAS 96-17-3), hexanal (CAS 66-25-1), 3-methylsulfanylpropanal (methional) (CAS 3268-49-3), benzaldehyde (CAS 100-52-7), 2-phenylacetaldehyde (CAS 122-78-1), 5-pentyloxolan-2-one (γ-nonalactone) (CAS 104-61-0), nonanal (CAS 124-19-6), (2*E*,4*E*)-nona-2,4-dienal (CAS 5910-87-2), ethyl hexanoate (CAS 123-66-0), (*E*)-4-(2,6,6-trimethylcyclohexen-1-yl)but-3-en-2-one (β-damascenone) (CAS 23696-85-7), methylsulfanymethane (dimethyl sulfide) (CAS 75-18-3), (methyldisulfanyl)methane (dimethyl disulfide) (CAS 624-92-0), (methyltrisulfanyl)methane (dimethyl trisulfide) (CAS 3658-80-8), butane-2,3-dione (diacetyl) (CAS 431-03-8). The internal standards, ethyl 2-methylpentanoate (CAS 39255-32-8), 4-fluorobenzaldehyde (CAS 459-57-4), 5-methyl-2-propan-2-ylcyclohexan-1-ol (menthol) (CAS 2216-51-5) and hexan-2,3-dione (CAS 3848-24-6) were supplied by Sigma-Aldrich (Germany). For the carbohydrate analysis, propane-1,2,3-triol (glycerol) (CAS 56-81-5), (2*S*,3*R*)-butane-1,2,3,4-tetrol (erythritol) (CAS 149-32-6), (2*S*,4*R*)-pentane-1,2,3,4,5-pentol (xylitol) (CAS 87-99-0), (2*R*,3*R*,4*R*,5*S*)-hexane-1,2,3,4,5,6-hexol (sorbitol) (CAS 50-70-4), (2*R*,3*R*,4*R*,5*R*)-hexane-1,2,3,4,5,6-hexol (mannitol) (CAS 69-65-8), glucose (CAS 50-99-7), fructose (CAS 57-48-7), saccharose (CAS 57-50-1), maltose (CAS 6363-53-7), maltotriose (CAS 1109-28-0), xylose (CAS 58-86-6), ribose (CAS 50-69-1), arabinose (CAS 10323-20-3), glycerol (CAS 56-81-5), and the internal standard 2-deoxy-D-glucose (CAS 154-17-6) were purchased from Sigma-Aldrich (Germany). For the analysis of the free amino acids lysine (CAS 56-87-1), aspartic acid (CAS 56-84-8), glutamic acid (CAS 56-86-0), asparagine (CAS 70-47-3), serine (CAS 56-45-1), glutamine (CAS 56-85-9), histidine (CAS 71-00-1), glycine (CAS 56-40-6), isoleucine (CAS 73-32-5), tyrosine (CAS 60-18-4), valine (CAS 72-18-4), methionine (CAS 63-68-3), tryptophan (CAS 73-22-3), threonine (CAS 72-19-5), alanine (CAS 56-41-7), arginine (CAS 74-79-3), phenylalanine (CAS 63-91-2), leucine (CAS 61-90-5), proline (CAS 147-85-3), and cell-free 5–100 mM amino acid mixture (98 atom-% ^13^C, 98 atom-% ^15^N) as internal standards were obtained from Sigma-Aldrich (Germany). 2-hydroxypropanoic acid (lactic acid) (CAS 79-33-4) and acetic acid (CAS 64-18-7) for analyzing the organic acids were purchased from Sigma-Aldrich (Germany). For the various eluents and sample dilutions, sodium hydroxide (CAS 1310-73-2), methanol (CAS 67-56-1), sodium dihydrogen phosphate dihydrate (CAS 13472-35-0), and acetonitrile (CAS 75-05-8) were acquired from VWR International (Darmstadt, Germany). Phosphoric acid (CAS 7664-38-2), acetic acid (CAS 64-18-7), and azanium acetate (ammonium acetate) (CAS 631-61-8) were purchased from Sigma-Aldrich (Germany). The total nitrogen analysis was performed using Kjeltabs Cu-3.5 from FOSS Analytical (Hilleroed, Denmark), sulfuric acid 95% (CAS 7664-93-9) and hydrogen peroxide (CAS 7722-84-1) from VWR International (Germany), sulfuric acid 0.05 M (CAS 7664-93-9) and sodium hydroxide 32% (CAS 1310-73-2) from neoFroxx (Einhausen, Germany), and 2-[[4-(dimethylamino)phenyl]diazenyl]benzoic acid (methyl red) (CAS 493-52-7) and 2,6-dibromo-4-[3-(3,5-dibromo-4-hydroxy-2-methylphenyl)-1,1-dioxo-2,1λ^6^-benzoxathiol-3-yl]-3-methylphenol (bromo-cresol green) (CAS 76-60-8) from Merck (Darmstadt, Germany). MRS (deMan, Rogosa, and Sharpe) agar ISO and MRS broth for microbial cultivations were purchased from Th. Geyer (Renningen, Germany).

### Lactic acid bacteria

The strains screened in this study were *Lacticaseibacillus rhamnosus* L1264 (DSM 20021) and *Lactiplantibacillus argentoratensis* L1276 (DSM 16365) obtained from the stain selections of the Chair of Microbiology (Technical University of Munich, Freising, Germany), and *Lactiplantibacillus plantarum* L758, *Lactiplantibacillus plantarum* L628, *Lactiplantibacillus plantarum* L879, and *Lactiplantibacillus plantarum* L762 from the Chair of Brewing and Beverage Technologies (Technical University of Munich, Freising, Germany). The strain identity was confirmed by MALDI-TOF (≥ 2.0) and 16S DNA Sequencing (≥ 99%) (compare supplementary information Table S1).

### Samples

The low-vicine/covicine faba beans *Vicia faba* var. TIFFANY were obtained from Norddeutsche Pflanzenzucht Hans-Georg Lembke KG (Holtsee, Germany). Sweet lupines *Lupinus angustifolius* var. BOREGINE were provided by Saatzucht Steinach GmbH & Co KG (Steinach, Germany).

### Substrate production

The germination of lupines and faba beans was performed according to Ritter, Gastl [9]. Briefly, seeds were soaked in water at 20 °C for 4 h (faba beans) or 3.5 h (lupines). Afterward, the seeds were placed in trays (2.4 kg per tray) and kept in a germination chamber (Viessmann, Allendorf, Germany) at 20 °C. To reach a water content of 52% and 62% for faba beans and lupines, respectively, further soakings for 10 min were performed on the consecutive four to five days. On the seventh day, germs were dried in a pilot malting plant for 24 h at 50 °C to obtain a storable malt.

The malt was ground in a laboratory mill ZM200 (Retsch, Haan, Germany) to pass through a 1 mm mesh and afterward mashed in a laboratory mashing device BMW12/CPU (Dinkelberg Analytics, Gablingen, Germany) to extract proteins and sugars and to obtain a liquid substrate for the subsequent fermentation. The mashing schedule contained a proteolytic rest at 45 °C for 30 min, a combined rest for phytic acid degradation and amylolysis at 60 °C for 30 min, and another combined rest for α-galactoside degradation and further amylolysis at 70 °C for 20 min. The commercially obtainable enzymes (all obtained from Novozymes, Lyngby, Denmark) were added to improve the enzymatic decomposition of major storage compounds further. Therefore, Neutrase 0.8 L BrewQ was added at 45 °C, and Ceramix Flex and Bio-Feed Phytase L were added at 60 °C. Finally, the mash was heated up to 80 °C and held for 20 min to reduce the seed-borne microbiological load. Afterward, the malt was separated by centrifuging (10 min, 4000 g, ambient temperature) in a Multifuge 4 KR centrifuge (Thermo Electron LED, Osterode, Germany) and subsequent filtration using folded filters (Macherey-Nagel, Düren, Germany). The resulting substrate was pooled and frozen until used in fermentation experiments.

### Design of experiment

A Box-Behnken design with 12 design points on the edges of the design space and five center points was chosen for the statistically-based fermentations. The Box-Behnken design is applicable to identify possible quadratic effects while keeping the parameters within their original ranges [37]. This seemed very important as a negative methionine addition resulting from a possible extension of a 2^3^ or 3^3^ factorial design towards a central composite design is not possible. However, as the impact of a methionine addition was to be studied in general, a minimum addition other than 0 mg/L would not have been applicable. The ranges of the parameters of the design space were tested to be appropriate in pre-experiments and are listed in Table 1. All experiments were performed in a random order to avoid falsification due to bias and possible lurking variables.

**Table 1:** Model parameter ranges of the experimental design

	model parameter		
variation	temperature T	inoculum I	methionine M
-1	25 °C	10∙10^6^ cells/mL	0 mg/L
0	33 °C	55∙10^6^ cells/mL	6 mg/L
1	41 °C	100∙10^6^ cells/mL	12 mg/L

After performing the statistically based fermentation experiments, the experimental design resulted in a model building for the individual response parameters (e.g., hexanal). First, a Grubb’s Test (α = 5%) for outliers was performed for the five center points in each response (e.g., hexanal). Linear (T, I, M), quadratic (T², I², M²), and interactive terms (T∙I, T∙M, I∙M) were considered for the model equations. Then, the parameters were reduced by forward stepwise regression utilizing the Bayesian Information Criterion (BIC) as the criterion to be minimalized. The coefficient of correlation (R²), the adjusted coefficient of correlation (R²adj), the Analysis of Variance (ANOVA) p-value, and the lack of fit were used to evaluate the quality of the models. Afterward, a few models were optimized by removing outliers using the Grubbs’ outlier test (α = 5%). Finally, the models were validated by performing additional experiments. Therefore, parameter set points within the design space but different from the original parameters were chosen to confirm the adequacy of the model predictions. These experiments were performed in triplicates on different days (with three biological replicates each day) to avoid falsification (e.g., due to a malfunction in the temperature control).

### Statistically based fermentations

Strains were taken from cryogenic storage and cultivated in MRS broth for one week before the screening experiments. Faba bean and lupine substrates were inoculated 1:50 (v/v) with MRS culture to adapt the strains to the legume substrates. Propagation was performed anaerobically but without forced degassing of the legume-based substrates prior to inoculation according to pre-experiments (data not shown). After 24 h at 28 °C, pH and optical density (600 nm) were measured to ensure proper growth and to calculate the required inoculum volume. This adapted culture was then used as inoculum for the fermentation experiments. For experiments in subsequent weeks, the MRS culture was re-inoculated in MRS broth for further use. Temperature, inoculum density, and methionine addition were chosen according to the experimental design. To avoid falsification due to the volume variation, if no/less methionine was added, 1 mL containing the appropriate methionine concentration (0–765 mg/L) was added. After 16 h, samples were taken to analyze volatile compounds and organic acids. To remove cells and to avoid further metabolic activities in the samples, those were centrifuged (5 min, 2800 g, ambient temperature) in a Universal 320 R centrifuge (Hettich Zentrifugen, Tuttlingen, Germany) and filtered using 0.45 μm dead-end filters (Macherey-Nagel, Germany). For the aroma analysis (HS-SPME arrow-GC-MS), the internal standard mixture was added before centrifuging and filtering. All samples were frozen and stored at -20 °C until further analysis. For the diacetyl analyses (GC-ECD), samples were directly frozen. Additionally, samples for the sensory evaluation were taken and stored at approx. 7 °C. Furthermore, pH and optical density (600 nm) were measured using a FiveEasy F20 pH meter (Mettler-Toledo, Greifensee, Switzerland) and Genesys 10 S UV-Vis photometer (Thermo Fisher Scientific, Dreieich, Germany), respectively. All fermentations were performed in biological triplicates. The model validation was performed as described for the original statistically-based fermentation but on three different days in biological triplicates to obtain statistically independent replicates.

### Bioreactor fermentations

To gain a deeper insight into time-dependent changes in the fermentation of legume-based substrates, fermentations with *Lactiplantibacillus plantarum* L628 in lupine-based substrate and *Lactiplantibacillus plantarum* L879 in faba bean-based substrate were performed in a Biostat B benchtop bioreactor (Sartorius, Goettingen, Germany). The bioreactor was equipped with a Dencytee RS485 225 cell density sensor (Hamilton, Bonaduz, Switzerland), an EasyFerm Plus PHI VP 225 pH sensor (Hamilton, Bonaduz, Switzerland), a Pt100 temperature sensor (Sartorius, Goettingen, Germany), and a pO_2_-sensor VisFerm DO ECS 225 (Hamilton, Bonaduz, Switzerland). Data acquisition was performed for the cell density sensor by using the software Dencytee arc Air version 3.8.1 (Hamilton, Bonaduz, Switzerland) and with SIMATIC SIPAT PCS7 (Siemens, Munich, Germany) for all other sensors. The bioreactor was temperature-regulated using a jacket heater and a WK 500 water bath (LAUDA Dr. R. Wobser, Lauda, Germany). Sampling was performed before and directly after inoculation, hourly for the first 12 h, and after 24, 32, and 48 h. Samples were taken and treated as described for the statistically-based fermentation. Furthermore, samples for the analysis of carbohydrates and amino acids were taken, centrifuged (5 min, 2800 g, ambient temperature) in a Universal 320 R centrifuge (Hettich Zentrifugen, Germany), filtered using 0.45 μm dead end filters (Macherey-Nagel, Germany), and frozen. In addition, samples from the substrate (prior to inoculation) and after 24 h of cultivation were spread on MRS agar plates. The substrate plates were examined after 5 and 8 days to confirm the absence of contaminations. The colonies on the 24-hour plates were counted after four days to evaluate the cell growth. The latter agar plates were also used for MALDI-TOF-MS confirmation of the lactobacillus.

### Chemical/Chromatographical analysis

#### Carbohydrate analysis

Soluble carbohydrates were analyzed using high-performance anion exchange chromatography with a pulse amperometric detector (HPAEC-PAD) on a Dionex ICS-5000 system (Thermo Fisher Scientific, Waltham, MA, USA) with a Dionex CarboPak PA10 analytical column (2 × 250 mm) and Dionex CarboPak PA10 guard column (2 × 50 mm) (both Thermo Fisher Scientific, USA). The mobile phases were (A) 0.250 M sodium hydroxide and (B) HPLC-grade water. The gradient was 20% A at 0 min, 20% A at 10 min, 90% A at 11 min, and 90% A at 19 min, followed by 10 min of equilibration at 20% A. The flow was set to 0.25 mL/min, and 1 µL was injected. The method was calibrated for the analytes glycerol, erythritol, xylitol, sorbitol, mannitol, glucose, fructose, mannose, arabinose, saccharose, ribose, maltose, and maltotriose obtaining regression coefficients of 0.99 or higher. Spiking experiments confirmed recovery rates of 84–107% (see Table 2 in the supplementary information). The limit of detection (LOD) and limit of quantification (LOQ) were determined by the signal-to-noise method (based on the peak height) described by Shrivastava and Gupta [38] (LOD = 3·S/N; LOQ = 10·S/N). Samples were prepared by defreezing, diluting with methanol, and mixing with the internal standard 2-deoxy-glucose. All analyses were performed in technical duplicates.

#### Organic acid analysis

The organic acids were measured by high-performance liquid chromatography with an ultraviolet detector (HPLC-UV) on an Ultimate U3000 system (Thermo Fisher Scientific, USA). The stationary phase consisted of a Synergi 4 μm Hydro-RP column (4.6 × 250 mm) with a C18 SecurityGuard Cartridge (4 × 3 mm) (both Phenomenex, Aschaffenburg, Germany). As mobile phase (A) 0.01 M sodium dihydrogen phosphate solution adjusted to pH 2.2 with phosphoric acid (85%) and (B) methanol was utilized. The gradient was 100% A at 0 min, 100% A at 10 min, 20% A at 14 min, and 20% A at 18 min, followed by 5 min of equilibration at 100% A. The flow was set to 0.7 mL/min, and 30 µL was injected. All analytes were measured at 210 nm. Calibration curves were obtained for lactic acid and acidic acid with regression coefficients of 0.99 or higher, and the recovery rates were all between 98 and 103% (see Table S3 in the supplementary information). The limit of detection (LOD) and limit of quantification (LOQ) were determined by the signal-to-noise method (based on the peak height) described by Shrivastava and Gupta [38] (LOD = 3·S/N; LOQ = 10·S/N). All analyses were performed in technical duplicates.

#### Amino acid analysis

The free amino acids lysine, aspartic acid, glutamic acid, serine, glutamine, histidine, glycine, isoleucine, tyrosine, valine, methionine, tryptophan, threonine, alanine, arginine, phenylalanine, leucine, and proline were analyzed according to Wannenmacher, Cotterchio [39] with modifications. Briefly, samples were diluted 1:40 and 1:1000 with 70% (v/v) acetonitrile, spiked with an isotope-labeled internal standard solution, and filtered through 0.20 μm dead-end membrane filters (Macherey-Nagel, Germany). The amino acids were analyzed using liquid chromatography with mass spectrometry (LC-MS) on an Agilent 1200 series HPLC system (Agilent, Waldbronn, Germany) coupled with a Triple Quad 4500 mass spectrometer (SCIEX, Darmstadt, Germany). Analytes were separated on an XBridge amide 3.5 μm 2 × 150 mm column with an amide security guard column (both Waters, Eschborn, Germany). The mobile phases were (A) HPLC-grade water with 7.5 mM ammonium acetate adjusted to pH 3 with acidic acid and (B) 95% (v/v) acetonitrile with 7.5 mM ammonium acetate adjusted to pH 3. The gradient was 5% A at 0 min, 5% A at 1 min, 10% A at 2 min, 10% A at 5 min, 30% A at 10 min, 40% A at 11 min, 40% A at 15 min, 5% A at 16 min, and 5% A at 19 min. The flow was set to 0.4 mL/min, and 2 µL were injected. The separated compounds were ionized by electron spray ionization (ESI). The device was calibrated using the amino acids lysine, aspartic acid, glutamic acid, serine, glutamine, histidine, glycine, isoleucine, tyrosine, valine, methionine, tryptophan, threonine, alanine, arginine, phenylalanine, leucine, and proline. All calibrations resulted in regression coefficients of ≥ 0.99 (compare Table S4 in the supplementary information). The limit of detection (LOD) and limit of quantification (LOQ) were determined by the signal-to-noise method (based on the peak height) described by Shrivastava and Gupta [38] (LOD = 3·S/N; LOQ = 10·S/N). All analyses were performed in technical duplicates.

#### Total and free nitrogen analysis

The total nitrogen in the supernatant was measured according to B-400.07.003 [40] with the Kjeldahl method, using an Kjeltec 8460 Analyze Unit with Sampler 8460, a Tecator Digestor Auto, and a Tecator Scrubber (all FOSS Analytical, Höganös, Sweden). To calculate the crude protein content, the conversion factor 5.4 was used for both legumes as recommended for edible legumes [41].

### Aroma analysis with head space solid phase micro extraction gas chromatography mass spectrometry (HS-SPME arrow-GC-MS)

The frozen samples containing the internal standard mixture were defrozen, 5 mL were transferred into a 20 mL glas vial, and immediately sealed airtightly using an aluminum cap. The 1.1 mm SPME arrow fiber 110 μm 20 mm (Thermo Fisher Scientific, Germany), coated with divinylbenzene/ carbon wide range/ polydimethylsiloxane (DVB/C-WR/PDMS), was conditioned at 40 °C for 1 min prior to analysis. Samples were placed in the cooled autosampler at 17 °C and preheated to 40 °C for 0.5 min, prior to extraction. Afterwards, the SPME fiber was exposed to the gas phase in the headspace of the vials for 30 min at 40 °C while shaking vigorously. After incubation, the volatile compounds were desorbed thermally in the GC system Trace 1310 (Thermo Fisher Scientific, USA) for 1 min at 250 °C and injected with a split ratio of 1:5 onto the low-polar TG-5MS column (length 60, inner diameter 0.25 mm, film thickness 0.25 μm; Thermo Fisher Scientific, USA). The flow rate of the carrier gas helium was 1.425 mL/min. The temperature of the GC oven was initially set to 60 °C and after 4 min the temperature was increased to 225 °C at a rate of 5 K/min and finally to 250 °C at 10 K/min, were it was hold for 4 min. The analytes were measured after being transferred into an ISQ OD mass spectrometer (Thermo Fisher Scientific, USA) set to EI mode in full scan mode (m/Z 35–350) and a dwell time of 0.2 s. All aroma analyzes were performed in technical duplicates. The aroma compounds of interest were identified in former studies [20, 33]. Verification of the aroma compounds was performed by comparing the retention time of the target compounds with the retention time of the respective analytical standard and the National Institute of Standards and Technology (NIST) Mass Spectral Library 2.0 database. Complex matrixes like the utilized legume-based substrates tend to retain or expel aroma compounds due to the interactions with further components (e.g., proteins). To compensate for such matrix effects, standard addition was chosen to calibrate the analytical method. Therefore, fermented substrates from multiple fermentations with different strains in lupine and faba bean substrates were pooled (for both substrates individually) and aroma standard mixtures in different concentrations were added. This was done, to compensate for the individual strain dependent changes of the matrix. A mixture of menthol, ethyl 2-methylpentanoate, and 4-fluorobenzaldehyde was used as internal standard for calibration and analysis. The calibration curves for the individual aroma compounds showed all correlation coefficients of ≥ 0.98 or higher (see Table S5 in the supplementary information). Limit of detection (LOD) and limit of quantification (LOQ) were determined by the signal-to-noice method (based on the peak height) described by Shrivastava and Gupta [38] (LOD = 3·S/N; LOQ = 10·S/N).

### Diacetyl analysis with head space gas chromatography electron captor detector (HS-GC-ECD)

Diacetyl (butane-2,3-dione) was measured according to B-420.21.157 [42]. Briefly, 4 mL sample were transferred in 20 mL glas vials mixed with 1 mL internal standard (hexan-2,3-dione) and sealed airtightly using aluminium caps. The vials were incubated for 90 min at 65 °C, cooled to ambient temperature and measured via automated head space sampling in a HP5890 Series II Plus gas chromatograph with electron capture detector (Hewlett-Packard Company, DE, USA) with HP7694 Headspace autosampler (Hewlett-Packard Company, Italy). The chromatographic parameters were 15 min vial equilibration at 65 °C, injection from the head space on a HP5 capillary column (length 30 m, inner diameter 0.32 mm, film thickness 1 μm, VWR International, Germany) with a split ratio of 1:15 and a gas volume flow (nitrogen) of 1.5 mL/min. After the isothermal separation (15 min at 50 °C), the analytes were detected in the ECD at 150 °C with hydrogen as detector gas (20 mL/min) and nitrogen as make-up gas (400 mL/min). The calibration curve for the diacetyl showed a correlation coefficient of 0.999, an LOD of 0.20∙10^− 3^ µg/L, and LOQ of 0.68∙10^− 3^ µg/L according to signal-to-noice method (based on the peak height) described by Shrivastava and Gupta [38] (LOD = 3·S/N; LOQ = 10·S/N).

### Matrix-assisted laser Desorption/Ionization – time of flight mass spectrometry (MALDI-TOF MS)

The colony forming bacteria on the 24 h samples of the bioreactor fermentations were confirmed by applying MALDI-TOF MS analysis. Therefore, several single colonies form the MRS agar were transferred onto MALDI plates (Bruker Daltonics, Bremen, Germany), and measured in a Microflex LT Spectrometer (Bruker Daltonics, Germany). Species were identified by spectra comparison with the Brucker Biotyper database (Bruker Daltonics, Germany).

*Sensory evaluation*.

The impact of the methionine addition on the aroma was evaluated by a sensory session, involving 16 trained panelists with former experience in sensory tests. Samples with and without methionine addition were presented in triangle tests. Moreover, in consecutive “rate all that applies” (RATA) evaluations the panelists were asked to rate the attributes “fruity/citrus/flowery”, “buttermilk/yogurt”, “beany”, “cooked vegetable”, and “sulfuric/potato” on a scale from 0 (not perceivable) to 5 (high intensity). Additionally, the overall hedonic rating was queried from 1 (not good) to 5 (very good).

#### Statistical analysis

Concentrations are given as mean values (± standard deviation) if not indicated otherwise. One-way analysis of variance (ANOVA) and Tukey’s HSD post-hoc test were used to statistically analyze data. All analyses were performed using JMP Pro 16 (SAS Statistical Discovery, NC, USA) and Origin 2018b (OriginLab Corporation, MA, USA).

## Results and discussion

In this section, first, the results of the statistically based fermentations are described. Second, the time-dependent changes observed in the bioreactor experiments are discussed with a deeper insight into the metabolic activities of the LAB. Prior to performing the experiments regarding the statistically based design, pre-experiments showed that all LAB employed in this study grow at the minimum parameters (results not presented). Therefore, the validity of the chosen temperature range was proven.

### Temperature had the highest impact on the aroma and taste active compounds in the statistically-based fermentations

The experimental design was supposed to unveil the impact of the fermentation parameters temperature, inoculum cell concentration, and methionine addition on various aroma compounds and the acetic/lactic acid ratio after fermenting lupine and faba bean-based substrates with several LAB strains (see Table 2). After the fermentation experiments, a total of 25 models were found for the different strains in lupine-based substrate (Table 3) and 23 for faba bean-based substrate (Table 4). In the validation, 88% of the models (22 out of 25) were confirmed for lupines and 74% (17 out of 23) for faba beans (*p* = 0.05). Some models show a statistically significant lack of fit (*p* < 0.05; see Tables 3 and 4), but were included as their model ANOVA p-value indicated a valid model [21]. A few model equations are listed in Tables 3 and 4, which did fail the ANOVA significance test but predicted the value of the respective aroma compound for the validation correctly. Nevertheless, those equations have to be interpreted with care.

**Table 2:** Target compounds for statistically-based fermentations and effect on refreshing beverages

Compound	Sensory description^1,2^	Effect on beverage	Fermentation target level
hexanal	green, grassy	detrimental	none–low
(*E*)-non-2-enal	fatty, green	detrimental	none–low
benzaldehyde	bitter almond, marzipan	beneficial	moderate
methional	cooked potato	detrimental	none–low
Dimethyl sulfides (DMS, DMDS, DMTS)	canned corn, sulfuric, cabbage	detrimental	none–low
ethyl hexanoate	fruity, pineapple	beneficial	high
β-damascenone	cooked apple, fruity, sweet	beneficial	high
γ-nonalactone	coconut, sweet	beneficial	high
diacetyl	buttery, sweet	beneficial	moderate
acetic/lactic acid ratio	pungent	detrimental	low

^1^aroma description according to Kreissl, Mall [24]

^2^effect of acetic/lactic acid ratio according to Rozada-Sánchez, Sattur [31], Peralta, Wolf [32], Nsogning Dongmo, Procopio [33]

**Table 3:** Model description for lupine-based substrate with model parameters, model quality and model validation

		model parameter^1^										model quality				validation
denominator	unit	Intercept	T	I	M	T∙I	T∙M	M∙I	T²	I²	M²	R²	R² adj	ANOVA	lack of fit	p-value^2^
		Lactiplantibacillus plantarum L628														
hexanal	µg/L	0.352	-0.204	-0.108	-0.066			0.176	0.199		0.120	0.797	0.662	0.0095	0.1212	0.5116
dimethyl trisulfide	µg/L	0.215	-0.048	0.001	-0.001			-0.070		0.038	-0.057	0.744	0.575	0.0240	0.0341	0.1308
(*E*)-non-2-enal	µg/L	0.013	-0.010	-0.009	-0.006			0.014		0.011		0.728	0.593	0.0118	0.0352	0.0832
γ-nonalactone	µg/L	1.243			0.017						-0.377	0.319	0.222	0.0680	not appl.	0.2260
β-damascenone	µg/L	0.702	0.764		0.120		0.201		0.377			0.906	0.874	< 0.0001	0.2291	0.6137
diacetyl	mg/L	0.196	-0.033	-0.058	-0.003						0.067	0.608	0.477	0.0169	0.0413	0.0989
acetic/lactic acid	-	0.052	0.007						0.016			0.395	0.309	0.0296	not appl.	0.5863
acetic acid	mg/L	489.7	100.6									0.350	0.307	0.0123	not appl	0.6967
lactic acid	mg/L	8939	601.3	525.6					-1249			0.694	0.623	0.0012	0.5474	0.0882
		***Lactiplantibacillus plantarum*** **L758**														
hexanal	µg/L	0.371	-0.278	-0.131		0.190			0.239			0.820	0.754	0.0004	0.0097	0.2240
methional	µg/L	0.605			0.193						0.235	0.345	0.251	0.0520	not appl.	0.8178
dimethyl trisulfide	µg/L	0.214		-0.031	0.024			-0.148				0.403	0.266	0.0735	0.0084	0.1985
ethyl hexanoate	µg/L	0.018		0.031	0.004			0.013		0.030		0.926	0.897	< 0.0001	0.3697	0.0036
(*E*)-non-2-enal	µg/L	0.014	-0.005		-0.003		-0.009		0.006			0.552	0.373	0.0678	0.0614	0.8584
β-damascenone	µg/L	0.989	0.762	0.115	0.193		0.277	-0.196	0.275		-0.234	0.945	0.902	< 0.0001	0.0991	0.1228
diacetyl	mg/L	0.336	-0.378	-0.130					0.317			0.799	0.749	0.0002	0.0392	0.3934
acetic/lactic acid	-	0.051	0.009	-0.006					0.019			0.605	0.514	0.0059	0.2098	0.9290
acetic acid	mg/L	496.2	90.0									0.468	0.433	0.0025	not appl.	0.7311
lactic acid	mg/L	9568	218.1	623.1		-752.5			-2096			0.861	0.814	< 0.0001	0.1727	0.3249
		***Lacticaseibacillus rhamnosus*** **L1264**														
hexanal	µg/L	0.422	-0.099	-0.075	-0.001	0.120	0.066	0.079				0.845	0.742	0.0031	0.2806	0.0028
ethyl hexanoate	µg/L	0.058	0.026	0.004	0.002	-0.023		0.025				0.765	0.658	0.0033	0.7964	0.6860
(*E*)-non-2-enal	µg/L	0.014	-0.005									0.155	0.099	0.1176	0.9239	0.5529
γ-nonalactone	µg/L	0.459			-0.251							0.344	0.298	0.0169	0.3887	0.3016
β-damascenone	µg/L	0.467	0.326	0.051	0.048	-0.119			0.160		-0.180	0.869	0.791	0.0006	0.9584	0.0537
diacetyl	mg/L	0.710	2.486	2.233	-0.074					2.168	2.140	0.750	0.624	0.0081	0.0073	0.0004

^1^The model parameters are T…temperature, I…inoculum, and M…methionine

The model equations can be set up by multiplying the model parameters with the constant terms in the table (e.g. hexanal, L628 = 0.352 − 0.204∙T-0.108∙I-0.066∙M + 0.176∙M∙I + 0.199∙T²+0.120∙M²)

^2^The results from the validation experiments were compared to the model prediction (*H*_0_: value_validation_ ≠ value_model_; *H*_1_: value_validation_ = value_model_). Therefore, high p-values indicate a good prediction of the result by the model

**Fig. 1:**
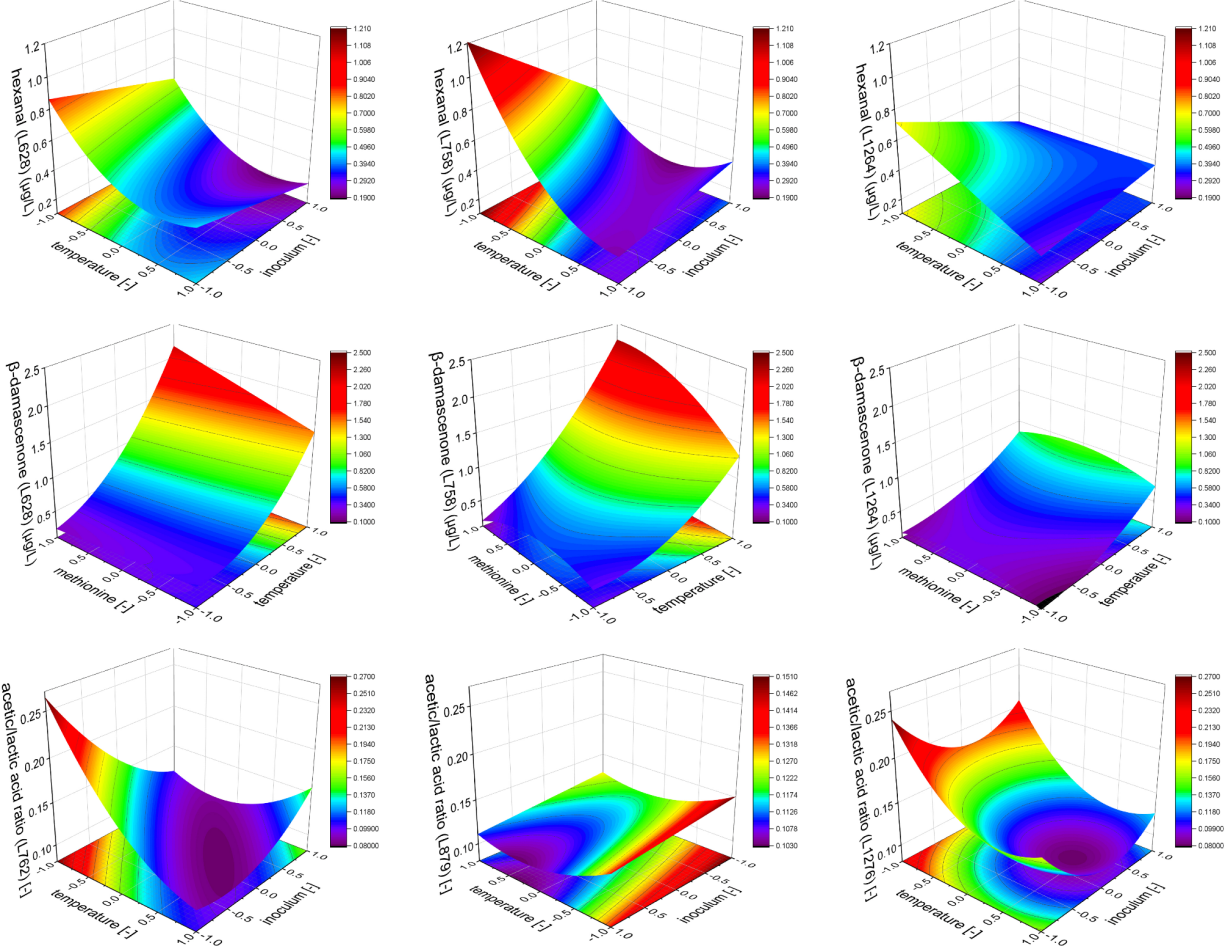
Depiction of the models exemplarily for hexanal (above) and β-damascenone (middle) in lupines and the acetic/lactic acid ratio (below) in faba beans. While the hexanal concentration after the fermentation was generally higher at lower fermentation temperatures, the magnitude of this characteristic was strongly strain-dependent. Higher inoculi had an impact at lower temperatures only. Likewise, the final β-damascenone concentration was highly temperature dependent, whereas higher temperatures resulted in higher concentrations. The ratio of acetic and lactic acid was shifted towards lactic acid with higher fermentation temperatures, except for L879 where the least changes were observed. The impact of the inoculum varied with the strain and the temperature. All graphs were drawn with a constant third parameter (above and below: 6 mg/L methionine addition, middle: 55∙10^6^ cells/mL inoculum cell concentration)

In lupines, the models for all strains show negative constant terms for temperature and inoculum regarding the final hexanal concentration (compare Table 3). This indicates that the final concentration of the aldehyde hexanal is lower at fermentations with elevated temperatures and higher inoculum cell concentration (see Fig. 1 above). At higher fermentation temperatures, the quadratic effect of the temperature acts as an attenuator (L628 and L758 only). Only the model for L879 in faba bean-based substrate contradicts this finding. However, in the validation, it was shown that the prediction of this model was significantly different from the actual result. As the aroma impression of hexanal is described as “green” and “grassy” [27] and connected to the beany aroma impression [29], its removal in the fermentation is highly preferable. Therefore, higher fermentation temperatures should be preferred.

The growth of the LAB is accelerated at higher temperatures, and higher inoculum cell concentration reduce its initial lag phase. Therefore, the reduced hexanal concentrations are likely due to a faster growth of the LAB and/or a faster acidification of the substrate. The ability of LAB to decrease the hexanal concentration is substantiated by several studies in soy [19, 43, 44], pea protein isolate [11, 45], and lupine protein [46]. Aldehydes are thereby utilized as electron acceptors via alcohol dehydrogenases to regenerate NADH to NAD^+^ [32, 47, 48], which yields alcohols like hexanol (from hexanal) or 2- and 3-methylbutanol (from 2- and 3-metylbutanal). Furthermore, aldehyde dehydrogenase might lead to the corresponding carboxylic acids of the respective aldehydes (e.g., hexanoic acid from hexanal) [16]. Comparable mechanisms can be assumed for the other aldehydes studied in the statistically based fermentations (benzaldehyde, (*E*)-non-2-enal, and methional).

**Table 4:** Model description for faba bean-based substrate with model parameters, model quality and model validation

		model parameter^1^																			model quality				validation
denominator	unit	intercept	T		I		M		T∙I		T∙M		M∙I		T²		I²			M²	R²	R² adj	ANOVA	lack of fit	p-value^2^
		Lactiplantibacillus plantarum L879																							
hexanal	µg/L	0.454	0.026		-0.021		-0.077		-0.068		0.103				-0.124					-0.127	0.969	0.944	< 0.0001	0.4721	0.0012
benzaldehyde	µg/L	0.269	0.066		-0.004		0.003		-0.032		0.071		-0.047		0.048		-0.068				0.847	0.694	0.0130	0.6237	0.0039
ethyl hexanoate	µg/L	0.032	-0.005		0.001		-0.003		-0.014		0.006		0.023				0.008			0.005	0.899	0.799	0.0028	0.1310	0.1316
β-damascenone	µg/L	0.924	0.688		-0.033		-0.032		-0.131		0.334		0.205		0.599						0.966	0.940	< 0.0001	0.9270	0.0034
diacetyl	mg/L	0.360	-0.263		-0.110				0.177												0.757	0.701	0.0003	0.0071	0.0836
acetic/lactic acid	-	0.113	-0.012		-0.008				0.018												0.543	0.438	0.0145	0.0052	0.4209
acetic acid	mg/L	751.1		36.0		-22.63				131.3											0.420	0.286	0.0623	0.1083	0.3359
lactic acid	mg/L	7114		940.8		158.5		-431.8		356.3		370.8		-514.8		-845		-255	208.7		0.984	0.964	< 0.0001	0.0720	0.0003
		***Lactiplantibacillus plantarum*** **L762**																							
dimethyl sulfide	µg/L	0.593	-0.141		-0.149										0.210						0.456	0.331	0.0421	0.3185	0.8293
benzaldehyde	µg/L	0.662	0.154		-0.029		0.007				0.194		-0.109		0.075					-0.149	0.821	0.682	0.0084	0.5132	0.3227
dimethyl trisulfide	µg/L	0.197	0.001		0.035		-0.014				0.087		-0.069								0.596	0.413	0.0480	0.9457	0.6071
(*E*)-non-2-enal	µg/L	0.011	-0.013		0.019		-0.011		-0.019				-0.028				0.018				0.867	0.787	0.0007	0.3154	0.4042
β-damascenone	µg/L	1.024	0.540		-0.066		-0.032				0.471				0.634		-0.183				0.964	0.942	< 0.0001	0.3197	0.0002
diacetyl	mg/L	0.452	-0.165		0.061		0.070		-0.080						-0.083		-0.104			0.116	0.794	0.634	0.0150	0.1216	0.2983
acetic/lactic acid	-	0.105	-0.030		-0.024				0.050						0.031		0.025				0.760	0.651	0.0036	0.0070	0.2711
acetic acid	mg/L	780.1		-80.8		-24.3		130.8		235.5								201.9			0.636	0.470	0.0295	0.0912	0.0711
lactic acid	mg/L	7343		575.8		737.6		449.4						712.8		-1522		490.0			0.866	0.786	0.0007	0.0106	0.0769
		***Lactiplantibacillus argentoratensis*** **L1276**																							
dimethyl disulfide	µg/L	0.640					0.312														0.326	0.281	0.0168	0.3574	0.1812
methional	µg/L	1.313	0.179		-0.292		-0.511		-0.385				0.394				-0.229				0.813	0.701	0.0034	0.3341	0.2764
β-damascenone	µg/L	1.218	0.405		0.275		0.360		0.396				0.719							0.477	0.864	0.783	0.0008	0.7159	0.0002
diacetyl	mg/L	0.590	-0.313																		0.308	0.262	0.0207	0.9704	0.7551
acetic/lactic acid	-	0.097	-0.041		-0.017										0.043		0.043				0.580	0.440	0.0246	0.0289	0.1425
lactic acid	mg/L	7271		820.6		193		207.4						-268.5		-1222		-381			0.919	0.870	< 0.0001	0.6677	0.0641

^1,2^ see Table 3

It needs to be mentioned that Shi, Hao [49] studied the impact of LAB fermentation on soymilk and soy protein solutions and found that hexanal can be bound to soy protein. Moreover, they reported that lower pH values and enzymatic protein hydrolysis could weaken the hexanal-protein bond, which was finally broken by a heat treatment after fermentation. This needs to be kept in mind as a possible subsequent pasteurization, which is typical for beverages to increase the shelf life, might release bound aldehydes and affect the aroma.

For strains fermenting faba bean-based substrate, two models were found to describe the final benzaldehyde concentration (L879 and L762). In both cases, higher temperatures increase the final concentration, while the inoculum cell concentration decreases it. Only for the methionine addition does the quadratic term in the model of L762 lead to a reduced predicted concentration at higher methionine values. However, it needs to be addressed that the model for L879 failed to predict the concentration in the validation. The presence of the typical “bitter almond, marzipan”-like aroma impression of benzaldehyde [27] in a final beverage might be beneficial at moderate concentrations, as it might suppress off-flavors, while high concentrations should be avoided.

The final yield of the aldehyde (*E*)-non-2-enal was temperature-dependent in all models, and while the first-order term was always negative, for L758 (in lupine-based substrate), a positive quadratic term was found. This indicates that higher fermentation temperatures lead to lower final (*E*)-non-2-enal concentrations, while this development might stagnate or even start to change at very high temperatures. The methionine addition consistently reduced the final (*E*)-non-2-enal yield, while the inoculum cell concentration was contradictive and led to an increase in L762 (faba bean) and a decrease in L628 (lupine). The very unpleasant aroma compound (*E*)-non-2-enal is described as “green” and “fatty” [27] and is known to trigger the aroma impression of “cardboard” in beer [30]. Therefore, its presence in a LAB-fermented beverage is not desired.

An opposite development can be seen for β-damascenone, where higher fermentation temperatures increase the final concentrations considerably in all models (compare Fig. 1). The impact of the inoculum cell concentration and methionine addition is less relevant for both legumes. Nsogning Dongmo, Sacher [50] used the same strain L758 as in this study for the fermentation of cereal-based substrates, compared its aroma profile with other LAB strains and found that L758 showed a distinctively high β-damascenone production. This supports our findings in lupine-based substrates, where the yield with L758 was slightly higher than L628 but considerably higher than L1264. However, it needs to be pointed out that the final β-damascenone concentrations in faba bean-based substrates were generally higher than in lupine-based substrates. This shows clearly that the strain, the fermentation parameter (mainly the temperature), and the substrate, are of utmost importance for the final β-damascenone yield. The ketone β-damascenone can be formed from the carotenoid neoxanthin by enzymatic or thermal degradation or by acidic oxidation [51]. The presence of the precursor neoxanthin was already reported for faba beans [52, 53] and zeaxanthin (a precursor for neoxanthin) was reported for lupines [54]. Most likely, the formation of β-damascenone in the LAB fermentation is due to several pathways. The bacteria-borne enzymes seem to have a strong influence as the resulting β-damascenone concentration is strain-dependent. However, Gijs, Chevance [55] observed elevated β-damascenone concentrations in a beer aging study at pH < 4.2 and 40 °C after aging for five days. Even as the time in this study was considerably less (only approx. 16 h), this might explain the principal influence of the temperature on the final β-damascenone yield as the fermentation conditions were comparable. Interestingly, in a statistically based fermentation with the yeast *Cyberlindnera saturnus* in a malt-based substrate at temperatures between 12 and 28 °C, the inoculum cell concentration was the main parameter, and the temperature as the subordinate term was slightly negatively correlated with the final β-damascenone concentration [21]. Importantly, no final pH value was below 4.7. This might further substantiate that a low pH is required, and a certain temperature needs to be reached to trigger a substantial β-damascenone formation. The aroma description of β-damascenone is “fruity,” “sweet,” and like “cooked apple” [27]. Therefore, and because of its very low odor threshold (0.0060 µg/kg in water [27]), it is very likely that β-damascenone can add a positive aroma impression to a beverage and might suppress less attractive odors.

For the sulfuric aldehyde methional, which is derived from the amino acid methionine and was added to a number of fermentations, only two models were found. With L758 in lupine-based substrate, the final concentration is exclusively connected to the methionine addition. However, the model for L1276 in faba bean-substrate is more complex and the addition of methional decreases the final methionine concentration according to the first order term. While this seems contradictory, it might be connected to the nutritive requirement of the LAB strain. The model also indicates a strongly reduced final methional concentration with higher inoculum cell concentration. This might indicate that methionine, which is only present in deficient concentrations in faba beans, is required for proper growth and vitality of the strain. Therefore, methionine would act as an important growth factor for the cell and could be utilized elsewhere in the metabolism instead of being converted to methional. Amárita, Fernández-Esplá [26] analyzed the methional production from methionine in 20 different LAB and found only one strain with the capability of producing higher amounts of methional, while four were found to produce low quantities. They concluded that the ability to produce methional from methionine is strain dependent. This confirms our findings and indicates that the selection of the LAB strain is of high importance for the final methional concentration.

The experimental data regarding the sulfuric aroma compounds dimethyl sulfide (DMS), dimethyl disulfide (DMDS), and dimethyl trisulfide (DMTS) only allowed the creation of a few models and should therefore be interpreted with care. The final DMS concentration decreased with higher temperatures and inoculum cell concentrations (L762, faba bean). For DMDS, only one model was found (L1276, faba bean). It describes increasing final DMDS concentrations with the addition of methionine and might substantiate the connection between the methionine addition and the formation of the sulfuric aroma compound. The impact of the fermentation parameters on the final DMTS concentration is rather complex and mainly characterized by the interaction terms. Consequently, the interpretation is not unambiguous. The final DMTS concentration was increased by high methionine additions (L758, lupine) and decreased by high inoculum cell concentrations (L758, lupine) and high temperatures (L628, lupine). However, contradictive results were found in L762 (faba beans) with high inoculum cell concentration, where a high methionine addition decreased the final DMTS concentration and a low methionine addition increased it. Regarding the metabolism, the amino acid methionine can be catabolized via the Ehrlich pathway to methional and degraded by γ-lyase via methanethiol to DMDS and DMTS [56]. In a subsequent reaction, DMDS can react to DMTS and DMS [57]. This is further backed by Lu, Fan [58], who studied the addition of methionine to the algae-containing water/sediment samples and found that it increases the concentration of DMS, DMDS, and DMTS.

A connection between the addition of methionine and disadvantageous increases in the sulfuric aroma compounds was not found unequivocally in this study. To further identify possible impairments of the overall aroma spectrum, a sensory analysis was performed. Neither was the addition of methionine significantly distinguished by the panel in lupine (*p* > 0.5) or faba bean (*p* = 0.45), nor showed the RATA analysis any significant differences for the attribute “sulfuric”. For the production of LAB-fermented beverages, this should be regarded as favorable as there is no evidence that the addition of methionine induces the production of aroma compounds with impressions like “potato,” “cabbaged,” or “sewer”. Consequently, the addition of the lacking amino acid methionine to equalize the amino acid spectrum in order to increase the bioavailability of the overall protein should be further researched.

The final diacetyl concentration was highly temperature-dependent with a slighter influence of the inoculum cell concentration for most strains. Generally, higher fermentation temperatures led to a lower diacetyl yield. The only exception in this study was *Lacticaseibacillus rhamnosus* L1264 (lupine), which produced very high diacetyl concentrations at higher temperatures and inoculum cell concentrations. L1264 was also the strain with the highest diacetyl yields in this study by far. On average, it produced 2.91 mg/L (± 3.25 mg/L) with a maximum of up to 10.35 mg/L (± 0.25 mg/L) compared with the 0.41 mg/L (± 0.30 mg/L) (average of the other five strains and all experiments). These analytical results were backed by the very intense “buttery” aroma impression of the samples fermented with L1264 at high temperatures. This contrary impact of the temperature on the diacetyl formation seems to be strain-dependent. For *Lacticaseibacillus rhamnosus*, the diacetyl production was reported to increase with the temperature up to 37 °C and decrease with further elevated fermentation temperatures [59]. However, Kim, Yoon [60] reported for two strains of *Lactococcus lactis* ssp. *cremoris* that the diacetyl production was highest at 20 °C and decreased with elevated temperatures. A comparable trend was stated for *Lactococcus lactis* ssp. *lactis* where higher temperatures led to a faster diacetyl production but with a lower final concentration [61]. A strain-dependent accentuation of the anabolism and catabolism of diacetyl might explain the reason for this inconsistent diacetyl development. First, this diketone can be formed via the α-acetolactate from pyruvate [32, 62] and alternatively from the amino acid metabolism of aspartic acid via α-keto acids [63]. Knowing that lupines and faba beans form very high concentrations of asparagine in the germination [9], this amino acid can be transformed to asperic acid by LAB-borne asperginases and hence further metabolized to diacetyl [63]. Once formed, diacetyl might be decomposed enzymatically by diacetyl reductase to acetoin and further to butane-2,3-diol [64, 65]. Depending on the ratio of diacetyl forming and degrading enzymatic activities of the particular strains, the reaction system leads to higher or lower final diacetyl yields. This is further substantiated by fermentation studies with *Lacticaseibacillus rhamnosus* [66] and *Lactococcus lactis* ssp. *cremoris* [60] reporting strong initial diacetyl increases followed by a decline after reaching a peak concentration. As the model for L1264 indicates, higher temperatures and higher inoculum cell concentrations lead to extremely high final diacetyl concentrations with this particular strain. This might be due to a deficit in the enzyme diacetyl reductase, which consequently leads to an accumulation of diacetyl.

Diacetyl as an aroma compound should be regarded equivocally. It might add a pleasant character of “yogurt” and “buttermilk” in lower concentrations, which could be an asset for LAB-fermented beverages. However, at higher concentrations, the aroma impression changes to be intensely “buttery” and not acceptable anymore. Therefore, the temperature and inoculum cell concentration are suitable parameters for adjusting the final diacetyl concentration. Nevertheless, the strain needs to be selected with care as some strains tend to yield unacceptably high diacetyl concentrations.

Only two poor models were found for γ-nonalactone (L628 and L1264, both lupine), which indicate a negative connection between the final yield of this lactone and high methionine addition. However, their predicted concentrations were confirmed in the validation and should, therefore, not be forfeited to hasty. No models were found for faba beans, as the γ-nonalactone concentration was mostly beneath the LOQ. The formation of γ-nonalactone can occur via four different reaction pathways from fatty acids, as reviewed by Romero-Guido, Belo [67]. For LAB in particular, increasing production of γ-nonalactone was reported in the fermentation of rice with *Lactobacillus paracasei*, and oleic acid and linoleic acid were identified as precursors [68]. As both legumes studied in this work are rich in those fatty acids, no limitation of precursors should be assumed. However, the production of γ-nonalactone might be strongly related to the choice of strain rather than to the fermentation parameters. Furthermore, the statistically-based fermentations were performed with CO_2_ as shielding gas to reduce the diffusion of oxygen into the fermentation broth. As the β-oxidation is a key reaction in one of the pathways, this might have reduced the γ-nonalactone formation and consequently could have led to lower yields. Romero-Guido, Belo [67] concluded for γ-decalactone that both the strain and the aeration are important for the production and that reducing the aeration first leads to lower yields because of insufficient NADH regeneration. However, the entire absence of oxygen ceases the accumulation of 3-hydroxy γ-decalactone and favors higher yields of γ-decalactone. In a beverage, γ-nonalactone is regarded as positive and might add a pleasant “coconut” aroma impression [27].

The final ethyl hexanoate yield was strongly increased with high inoculum cell concentrations in combination with high methionine additions (L758 and L1264, both lupine). Whereas, the inoculum had no or a slightly adverse effect on the ethyl hexanoate yield without or at low methionine additions. However, only few models were found in general and especially for L879 (faba bean) very complex.

Therefore, the production of ethyl hexanoate seems to be highly strain-dependent and not easily explainable by the fermentation parameters alone. In general, ethyl hexanoate, among other compounds (e.g., hexyl hexanoate), is formed from the esterification of hexanoic acid and an alcohol (compare Ritter, Gastl [15]). Consequently, the tendency to react hexanal towards hexanoic acid and further towards esters or alternatively to accumulate hexanol seems to depend on the individual strain. The connection between hexanal and ethyl hexanoate can be seen in the model of L1264, where increasing ethyl hexanoate yield go along with reduced hexanal concentrations. The aroma impressions of “fruity” and “pineapple” [27], triggered by ethyl hexanoate, could improve the overall aroma of a beverage.

The production of lactic and acetic acid is influenced mainly by the temperature and the inoculum cell concentration. For most strains, elevated fermentation temperatures (at comparable values for methionine (M = 1) and inoculum (I = 1)) lead to increasing lactic acid yields until an optimum was reached, while the acetic acid yield was increasing linearly. This lead to a reduced acetic/lactic acid ratio with increasing temperatures up to an optimum value from which the ratio started to increase again. Therefore, two conclusions can be drawn. First, the lactic and acetic acid production are highly temperature dependent, and second, while a temperature optimum can be found for the final lactic acid yield, the acetic acid yield increases continuously with the temperature. Bassit, Boquien [61] reported for *Lactococcus lactis* subsp. *lactis* in a diary application that the lactic acid production doubled by elevating the fermentation temperature from 18 to 30 °C. Moreover, even as the maximum population of LAB was identical after a fermentation time of 24 h, about 25% more lactic acid was produced. They also revealed that for the particular strain, there was a shift from diacetyl production towards lactic acid production with increasing fermentation temperatures. For sourdough, increasing lactic and acetic acid concentrations with higher temperatures were reported, but no optima of their ratio was found [69]. Consequently, in the production of LAB fermented beverages, the temperature needs to be chosen carefully to create a well-balanced acidity and to avoid the pungent taste impression of acetic acid.

The production of acidic acid is commonly connected to the metabolism of heterofermentive LAB. In difference to homofermentative LAB, where pentoses are metabolized to lactic acid via the pentose phosphate pathway, heterofermentative LAB for lactic and acidic acid via the phosphoketolase pathway [32]. However, in the presence of electron acceptor, pyruvate (e.g., from glucose) can be metabolized via acetyl-phosphate to acidic acid. This is energetically beneficial as two additional ATP are formed. For the required regeneration of the cofactor NADH to NAD^+^, aldehydes, phytochemicals, oxygen and/or hydrogen peroxide are reduced enzymatically by NADH oxidase or peroxidase [16, 32, 48, 70]. Additionally, under aerobe conditions, acetate is produced together with diacetyl and acetoin from pyruvate [32]. Therefore, even as all strains used in this study are categorized as homofermentative (according to Zheng, Wittouck [71]), the presence of acidic acid is very plausible.

Comparing the different models for an individual strain, it becomes evident that shifts of a model parameter in one direction does not improve all response variables. Therefore, finding optimal fermentation parameters is a challenging task and always involves a compromise. For the strains *L. plantarum* L628 (in lupine substrate) and *L. plantarum* L879 (in faba bean substrate), optima were named, which were used for the validations of the statistical models and the bioreactor fermentations. Those were 38 °C (0.73), 73∙10^6^ cells/mL (0.4), and 8.4 mg_methionine_/L (0.4) for L628 and 39 °C (0.86), 96∙10^6^ cells/mL (0.9), and 11.4 mg_methionine_/L (0.9) for L879. The values in brackets indicate the respective corresponding values in the design space. These parameters were chosen to archive low concentrations of hexanal, (*E*)-non-2-enal, and DMTS and moderate concentrations of diacetyl while increasing the yield of γ-nonalactone, β-damascenone, and ethyl hexanoate. Moreover, the ratio of acidic to lactic acid was tried to be low to diminish the pungent taste impression and the vinegar-like odor of acidic acid.

### Time-dependent changes in the LAB fermentation of legume-based substrates

**Fig. 2:**
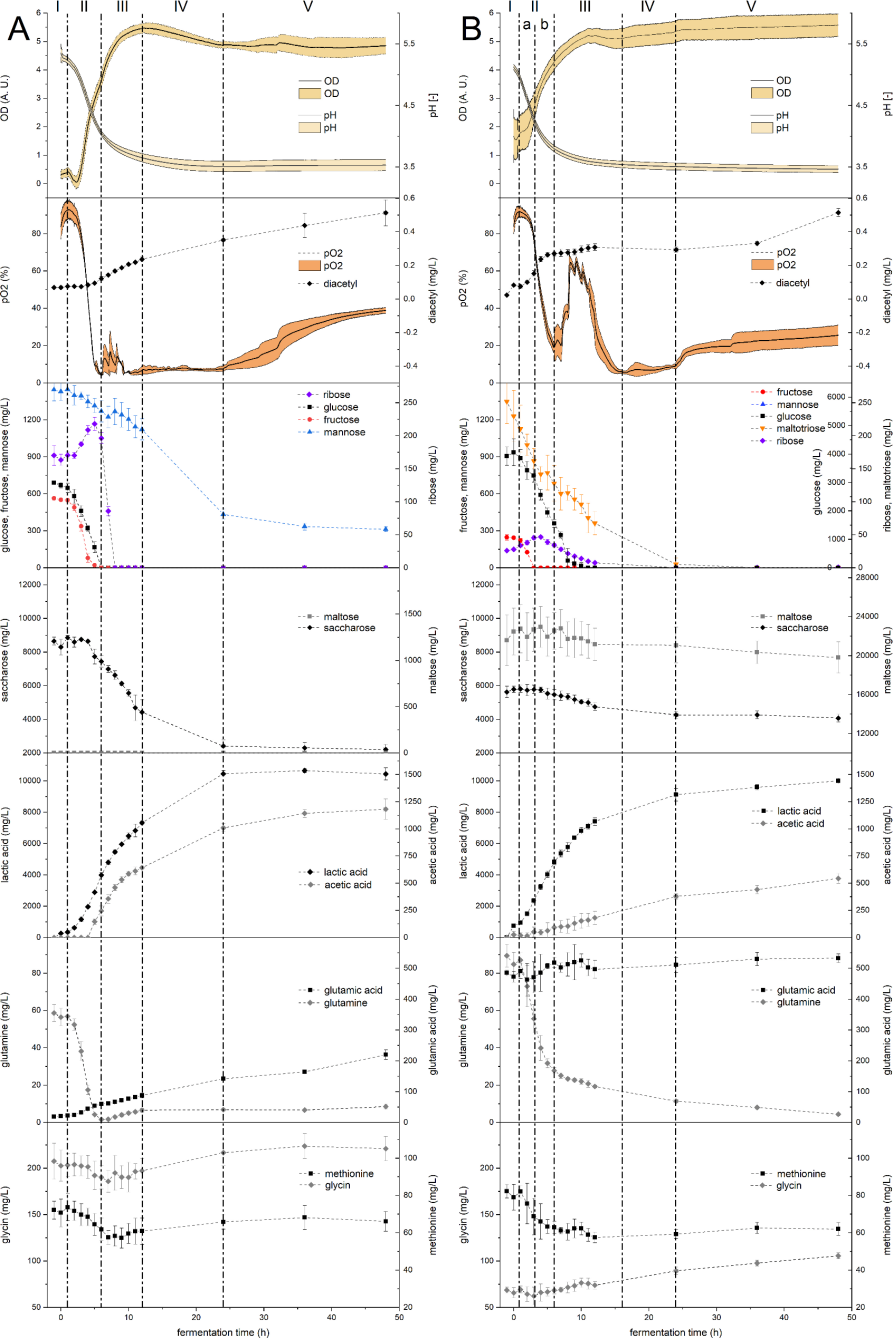
Changes in the bioreactors with *Lactiplantibacillus plantarum* L628 in lupine- (A) and *Lactiplantibacillus plantarum* L879 in faba bean-based substrates (B) over the 48 h of fermentation. The time “-1 hour” represents the measurement of the substrate prior to inoculation, and “0 hours” directly after inoculation. The diagrams show from top to bottom: 1, optical density (inline) and pH (inline); 2, diacetyl and oxygen partial pressure (inline); 3, carbohydrates ribose, glucose, fructose, mannose, and maltotriose (faba bean only); 4, carbohydrates maltose and saccharose; 5, organic acids lactic and acetic acid; 6, amino acids glutamine and glutamic acid; 7, amino acids methionine and glycine. Please keep in mind that for glucose and maltose, the scale of the axis differs between lupine and faba bean. Abrupt changes in the fermentation behavior are indicated with a vertical dotted/dashed line and numbered with Latin numbers. (*n* = 4)

The LAB grew well in all bioreactor experiments. After 24 h of fermentation, L628 reached 9.21 (± 0.03) log_10_ CFU/mL in lupine- and L879 grew to 9.13 (± 0.07) log_10_ CFU/mL in faba bean-based substrates. In all fermentations, the colonies on the MRS agar plates showed a uniform appearance, and the strain identity was successfully confirmed via MALDI-TOF (compare Table S6 in the supplementary information). Furthermore, no colonies were detected on the MRS agar plates treated with the substrate samples taken prior to inoculation. Therefore, a possible contamination can be excluded. Figure 2 presents an overview of the changes in the optical density, pH, dissolved oxygen (pO_2_), carbohydrates, organic acids, and selected amino acids in the bioreactor fermentations and compares the developments in lupine- and faba bean-based substrates. The adaption of the LAB in both substrates was fast, and microbial growth set in after approx. 2.5 h (lupine) and 2.0 h (faba bean). In lupines, two different exponential growth phases were identified with specific growth rates of µ_1_ = 0.466 h^− 1^ and µ_2_ = 0.150 h^− 1^, but only one with µ_1_ = 0.338 h^− 1^ was identifiable doubtlessly in faba beans. However, another growth phase is highly likely as explained below, but not distinguishable due to fluctuations.

The fermentations can be divided into five different growth or metabolic sections (marked with Latin numbers and separated with vertical dotted/dashed lines in Fig. 2). In both legumes, the lag phase (section I) lasted until growth set in at approx. 2 h. In this first section, the LAB adapted to the new environment, and no considerable changes were observable.

Section II (2–6 h in lupine, 2–7 h in faba bean) marked the exponential growth phase. This can be clearly seen in the substantial decrease of the dissolved oxygen, the pH-drop, and the sharp increase of the optical density. Simultaneously, in lupine, fructose and glucose were metabolized while the ribose concentrations increased. In faba beans, section II is divided in two sub-phases. First, ribose increased, fructose was entirely depleted, and diacetyl started to be formed (a) and second, ribose started to decrease (b). While fructose and glucose are common degradation products of the seed’s carbohydrates saccharose, raffinose, stachyose, and verbascose (S. Ritter et al., 2023), ribose was not mentioned in connection with lupines or faba beans so far. However, for several other legumes (lentils, dry peas, white beans, pinto beans, and chickpeas), small concentrations were reported to appear in the seeds [72]. Interestingly, even as ribose is not a cleavage product of the initially available carbohydrates, the ribose concentration increases significantly by 50.6 mg/L in lupine- and 20.5 mg/L in faba bean-based substrates within the first hours of the fermentation. For several bacteria of the genera *Bacillus*, the production of ribose as a by-product in the metabolism of glucose via the glycolysis and pentose phosphate pathway was reviewed by Wulf and Vandamme [73]. As LAB also possess the ability to utilize the same metabolic pathways [32], the small amounts of ribose were probably formed by comparable mechanisms. In the amino acids, a very strong drop of the glutamine concentration can be observed in both legumes, while it reached a minimum in lupines and was continually decreasing in faba beans. The glutamic acid concentration slightly increased in lupine and fluctuated in faba beans, while methionine decreased slightly in both legumes.

Strong fluctuations in the dissolved oxygen after reaching a first minimum marked section III. In lupine, the optical density indicated a second exponential growth phase, before the growth decelerated as well as in faba bean. While the production of diacetyl stagnated in faba beans, it continued in lupine. Ribose was depleted in both legumes, whereas its utilization was considerably stronger in lupine. Moreover, glucose was depleted in faba beans, while mannose (which was not observed in faba beans) and saccharose were metabolized in lupines. Notably, the changes in the maltose concentration (only faba beans) were not statistically significant. However, as the concentration of maltotriose decreased strongly, it is most likely that an equilibrium between metabolic maltose utilization and maltose replenishing maltotriose decomposition existed in the observed timeframe of the fermentation experiments. A turning point in the amino acid glutamine was clearly perceivable, in lupine, while it continued to decrease in faba beans and methionine began to increase slightly in both legumes.

During section IV (starting after 12 h in lupine and after 16 h in faba bean), the dissolved oxygen remained at a very low level and the pH reached its final value. In this phase, the concentration of the last remaining carbohydrates (mannose and saccharose in lupine and maltotriose in faba bean) decreased, while the further trends continued as described for section III.

After 24 h, the stationary phase (section V) was reached in both legumes. The values for the optical density, the pH, and most amino acids remained stable. In the dissolved oxygen, a renewed increase was perceivable, which indicates less consumption of oxygen, e.g., as electron acceptor. While the concentrations of the organic acids stagnated in lupine, they continued to increase slightly but statistically significant in faba bean. Interestingly, the concentration of diacetyl continued to increase in lupine and even accelerated in faba beans.

Both L628 (lupine) and L879 (faba bean) are homofermentative, facultatively anaerobe LAB [71]. However, the dissolved oxygen decreased rapidly with the exponential growth. At the same time, the production of acetic acid and diacetyl set in, which indicates that oxygen was utilized as an electron acceptor to regenerate NADH to NAD^+^. This alternative utilization of pyruvate with acetic acid, diacetyl, and acetoin as metabolic products is known for homofermentative LAB under aerobic conditions [32]. This is supported by the total absence of mannitol in the fermentation, which is usually formed by heterofermentative LAB when using fructose as an electron acceptor in the regeneration of NADH to NAD^+^ to reroute the phosphoketolase pathway from ethanol towards acetic acid as the end product [32].

In both strains, the glutamine concentration strongly decreased within the first 6 h of fermentation. However, while the glutamic acid concentration increased in L628 (lupine) continually, it fluctuated but remained at the same level in L879 (faba bean). For L628 this can be explained with a conversion of glutamine into glutamic acid to enhance acid tolerance by LAB. Therefore, glutamate is intracellularly deamidated, and the resulting glutamic acid is released into the substrate, where it accepts protons and stabilizes the pH value [74]. Additionally, glutamine can be converted to γ-aminobutyric acid [75], which would explain the missing increase of glutamic acid in L879 (faba bean). In L628 (lupine), the accumulation of glutamic acid did not stop with the depletion of glutamine but continued even at a slightly slower pace. At the same time, the glutamine concentration started to increase as well. This might be explained by the further activity of proteolytic enzymes releasing free glutamine from legume protein and the release of intracellular amino acids due to starting cell lysis. This assumption is backed by the slightly increasing concentrations of the majority of free amino acids (compare Table S7 and S8 in the supplementary information). A further mechanism to increase acid tolerance would be the conversion of arginine to ornithine [76]. However, reductions in the arginine concentration were neither observed in L628 nor L879 (see Table S7 and S8 in the supplementary information). The sum of free amino acids did not change statistically significantly in the fermentations.

The diacetyl production was strong at the beginning of the fermentations but continued in the stationary phase in L628 (lupine) and even increased in L879 (faba bean). Diacetyl, as a metabolic product, is favored by low pH values and low carbohydrate concentrations and under aerobe conditions [77]. This might explain especially the increasing diacetyl concentrations at the end of the fermentations with L879 (faba bean). Once the monosaccharides were depleted, the pH approached its final value of approx. pH 3.5, and the dissolved oxygen started to increase again. Hugenholtz, Kleerebezem [62] observed a shift from complete homolactic fermentation under anaerobic conditions towards a mixed acid fermentation (75% lactic acid, 18% acetic acid) under aerobic conditions in *Lactococcus lactis*, whereas diacetyl was formed under aerobe conditions exclusively. This difference was even intensified in a knockout mutant, where the lactic acid production was shifted entirely towards acetic acid production in combination with 16% of glucose being converted to diacetyl. Regarding the time-dependent development of the diacetyl concentration in the lactic acid fermentation, very different findings were reported. Such are continually increasing diacetyl concentration until the end of fermentation [65], relatively stable diacetyl concentrations after a substantial increase within the first hours [78], or even decreasing concentrations after reaching a peak [60].

Diacetyl itself is formed via chemical oxidation from α-acetolactate and can be converted to acetoin by diacetyl reductase and further to butane-2,3-diol by acetoin reductase [64]. Consequently, fermentations with strains showing a strong diacetyl reductase activity would result in a lower diacetyl concentration in the stationary phase. Contrarily, in fermentations using strains without or with low diacetyl reductase activity, higher concentrations of diacetyl would remain. Importantly, no decrease of lactic acid in combination with an increase of acetic acid was perceivable. This would be observable if the strains would start to metabolize lactic acid into acetic acid to maintain the pH value in aerobe conditions under glucose starvation, as observed in *L. plantarum* by Goffin, Lorquet [79].

### Aroma compounds change considerably with the progressing LAB fermentation

Fermentation highly changed the aroma spectrum in both legumes. As aroma compounds are perceived highly differently, those changes are discussed in the context of the respective odor thresholds (in water), which were all retrieved together with their odor impression from the Odorant Database of Kreissl, Mall [27]. However, the odor threshold should be regarded more as an indication instead of a fixed value as odor perception varies highly from person to person, with the degree of the person’s training and even for the same person from day to day.

**Fig. 3:**
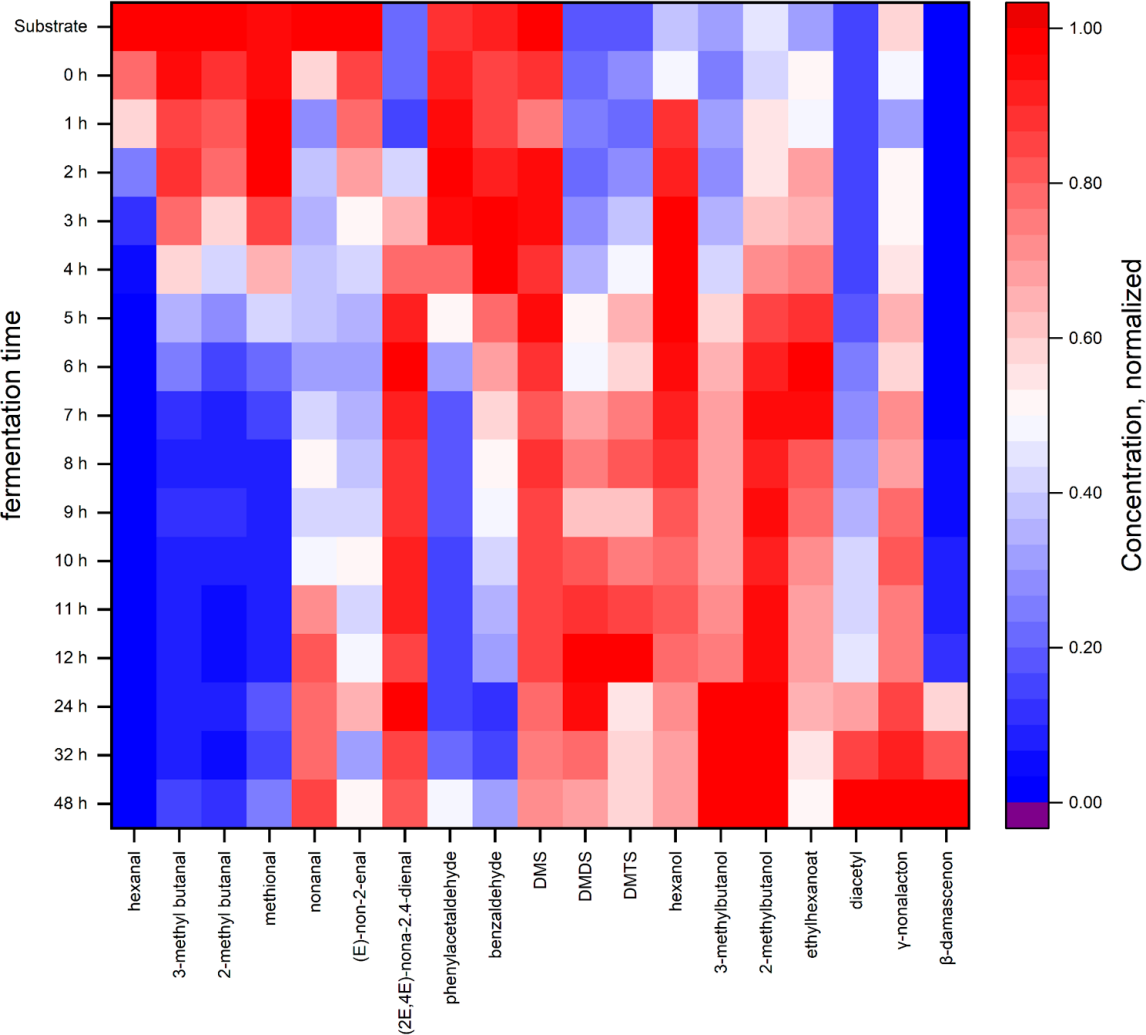
Development of aroma compounds in the fermentation of lupine-based substrate with *Lactiplantibacillus plantarum* L628. To depict changes for all compounds despite their highly different concentration levels, the concentrations were normalized by dividing by the respective maximum values. (*n* = 4)

In lupines, the aldehyde hexanal almost disappeared within the first 4 h (compare Fig. 3). Its concentration dropped from 24.4 ± 0.94 µg/L in the substrate to 1.52 ± 0.10 µg/L, which is beneath the reported odor threshold (2.4 µg/L). Comparable decreases were observable in other aldehydes, too. In methional (cooked potato-like), 2- and 3-methylbutanal (both described as “malty”), concentrations dropped to approx. 10% of the initial value within 8 h. Thereby, 3-methylbutanal and methional did not drop to concentrations beneath their odor threshold of 0.5 µg/L and 0.43 µg/L, respectively, at any point of the fermentation. Benzaldehyde (“bitter almond”, “marzipan”) and phenylacetaldehyde (sweet, floral, and honey-like) started to decline after an initial incline within the first 3–4 h. While the concentration of benzaldehyde was far beneath its odor threshold (150 µg/L) during the entire fermentation, phenylacetaldehyde declined beneath its odor threshold of 5.2 µg/L and increased above it to 12.1 ± 2.11 µg/L after 48 h. The rather unpleasant aldehydes nonanal (“soapy”, “green”, “oily”, “citrus”) and (*E*)-non-2-enal (“green”, “fatty”, “cucumber”) seem to decrease initially and started to increase slightly after approx. 6 h. However, neither of them reached concentrations above their odor thresholds of 2.8 µg/L and 0.19 µg/L, respectively. (2*E*,4*E*)-nona-2,4-dienal (“green”, “fatty”) even increases strongly in the first 6 h of fermentation from 0.013 ± 0.003 µg/L to 0.060 ± 0.005 µg/L, which was slightly above the odor threshold of 0.046 µg/L and remained stable afterward. Most of the aldehydes seem to increase slightly at the end of fermentation (last 24–48 h). The corresponding alcohols hexanol (to hexanal), 2-methylbutanol (to 2-methylbutanal), and 3-methylbutanol (to 3-methylbutanal) increased as the aldehydes decreased. With the exception of hexanol, they stayed at a high level until the end of fermentation. Kaseleht, Paalme [47] proposed that homo- and heterofermentative LAB utilize aldehydes as electron acceptors to regenerate NADH to NAD^+^ while forming the corresponding alcohol. This explains the substantial reduction of the aldehydes in combination with the increase in the alcohols. The hexanol concentration decreased after reaching a maximum of approx. 3–5 h, which might be due to the formation of hexanoic acid. As the odor threshold of alcohols is by magnitudes higher than the one of their corresponding aldehydes (e.g., 590 µg/L for hexanol and 2.4 µg/L for hexanal [27]), the conversation of aldehyde to alcohol can highly reduce off-flavors. However, as this reaction is reversible, a probable increase of the corresponding aldehydes during the storage period should be monitored. The sulfuric compound DMS (“canned corn”, “cabbage”) showed only slight but not significant changes in the fermentation and remained with 16.1 µg/L (end of fermentation) to 22.2 µg/L (unfermented substrate) higher than its odor threshold of 0.30 µg/L. The related compounds DMDS (“cabbage”, “putrid”) and DMTS (“cabbage”, “sulfuric”) increased in the first 12 h of fermentation and decreased slightly but significantly afterward. At the end of the fermentation DMDS was with 0.27 ± 0.14 µg/L beneath its odor threshold of 1,7 µg/L and DMTS was with 0.14 ± 0.11 µg/L above the very low threshold of 0.0099 µg/L. The unpleasant sulfuric compounds are formed as degradation products of the sulfuric amino acids methional and cysteine [57, 80].

Ethyl hexanoate (“fruity”, “pineapple”) increased within the first 6–7 h and decreased afterward, but with 0.0068–0.0205, µg/L never exceeded its odor threshold of 1.2 µg/L. The fruity and coconut-like γ-nonalactone increased from 0.57 ± 0.09 µg/L in the substrate and reached 0.98 ± 0.11 µg/L after 48 h and, therefore, remained clearly beneath its odor threshold of 9.7 µg/L. However, the very pleasant β-damascenone (“fruity”, “cooked apple”, “honey”) increased almost exponentially from 0.017 ± 0.002 µg/L to 4.50 ± 0.84 µg/L during the fermentation, which exceeded the odor threshold of 0.006 µg/L by magnitudes. The diketone diacetyl, which is described as buttery and sweet and can introduce a pleasant buttermilk-like aroma, also showed considerable increases of 0.07 ± 0.00 mg/L to 0.51 ± 0.08 mg/L, which was firmly above its threshold of 1 µg/L.

**Fig. 4:**
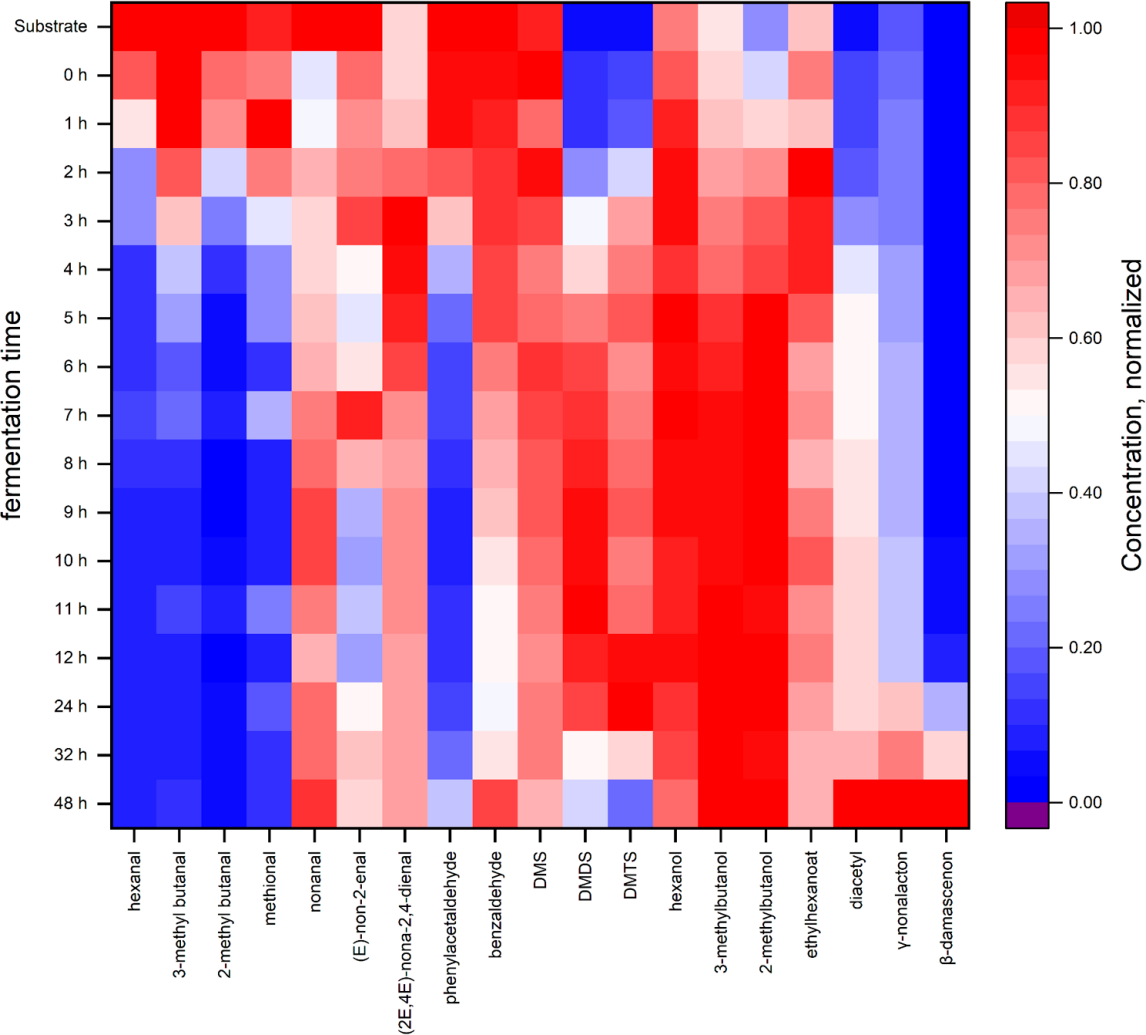
Development of aroma compounds in the fermentation of faba bean-based substrate with *Lactiplantibacillus plantarum* L879. To depict changes for all compounds despite their highly different concentration levels, the concentrations were normalized by dividing by the respective maximum values. (*n* = 4)

In faba beans, the changes were comparable for most compounds (Fig. 4). A different development was observed for (2*E*,4*E*)-nona-2,4-dienal, which did not increase as in lupines but fluctuated slightly. While in lupines, concentrations up to 0.060 ± 0.005 µg/L were measured, negligible amounts between 0.0029 µg/L and 0.0047 µg/L were found. It needs to be mentioned that all measurements of (2*E*,4*E*)-nona-2,4-dienal remained beneath the LOQ and by a magnitude beneath the odor threshold of 0.046 µg/L. γ-nonalactone did not seem to reach a limit as in lupines but increased linearly. However, the final concentrations remained at 0.37 ± 0.05 µg/L, which was far beneath the 0.98 ± 0.11 µg/L in lupines. Strikingly, the initial hexanal concentration in lupines (24.4 ± 0.95 µg/L) was 16-fold higher than in faba beans (1.50 ± 0.08 µg/L). However, upon fermentation, both concentrations decreased and were comparably low after approx. 7 h. This more substantial decrease in hexanal can also be seen in the corresponding alcohol. In lupines, the initial hexanol concentration was 41.5 ± 0.76 µg/L, ten times higher than in faba beans (4.03 ± 0.22 µg/L). While its concentration in faba beans changed only slightly, it increased up to 105.3 ± 5.51 µg/L (4 h) before it declined to 71.1 ± 6.94 µg/L (48 h). Comparably, the benzaldehyde concentration was 14 times higher in lupines (11.9 ± 0.17 µg/L). As both were reduced in the fermentation, the resulting concentrations after fermentation were only approx. 5-times higher in lupines (4.03 ± 0.74 µg/L compared with 0.74 ± 0.09 µg/L in faba beans). Contrary to lupines, the diacetyl production seemed to vary in faba beans with the fermentation time. There was an initial increase in the first 4 h, followed by a stagnation of slight increase and a final incline from 0.33 ± 0.01 µg/L to 0.51 ± 0.02 µg/L in the last 12 h. The stagnation coincides with an increase in the dissolved oxygen starting approx. 6 h after inoculation (see Fig. 2). Further differences in the absolute concentrations were found for DMS, where lupines showed 3–4 times higher concentrations than faba beans. In faba beans, the initial 2-methyl butanal concentration was 2.51 ± 0.11 µg/L, four times higher than in lupines. However, the concentrations approximate during the fermentation.

Generally, in both legumes, the negatively associated aldehydes hexanal, 2- and 3-methylbutanal decreased strongly while their corresponding alcohols increased. The sulfuric aldehyde methional decreased, but other sulfuric aroma compounds increased. Most conveniently for beverage applications, aroma compounds with fruity and buttermilk-like aroma impressions increased strongly.

### Overall comparison of microbial induced changes of legume-based substrates

**Fig. 5:**
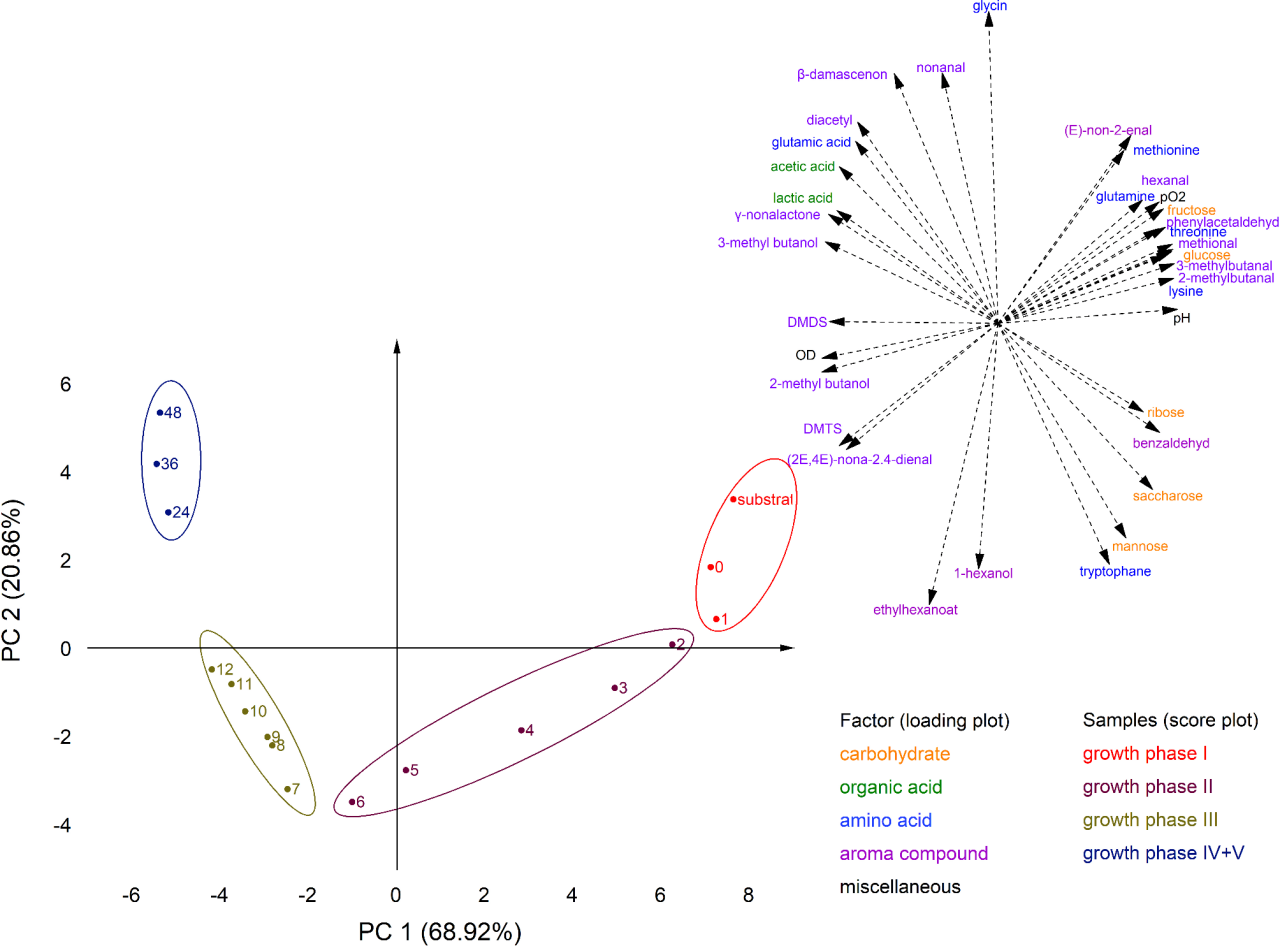
Principal Compound Analysis of all observed statistically significant changes in the fermentation of lupine-based substrates with *Lactiplantibacillus plantarum* L628. The score plot (left) shows the samplings at different times in the fermentation. The distance between the data points of 2–6 h indicate very pronounced changes in this growth phase. In contrast, the data points 7–12 h are clustered closely together, indicating less pronounced changes. The loading plot (right) describes the 35 influencing factors from the group’s carbohydrates, organic acids, amino acids, aroma compounds, and miscellaneous (like the pH value). To depict changes for all compounds despite their highly different concentration levels, the concentrations were normalized by dividing by the respective maximum values. PC 1 and PC 2 explain 89.8% of the total variation. (*n* = 4)

The Principal Compound Analysis (PCA) of the fermentation of lupine-based substrate presents all data of statistical significance (Fig. 5). It confirms that changes are of varying profoundness depending on the growth phase in the LAB fermentation (Fig. 5). Whereas Fig. 2 indicated no further changes in the first hour of the fermentation, the PCA shows unequivocal changes. In growth phase II, the most pronounced differences appear, which can be seen in the elevated distance between the data points. In contrast, the data points of growth phase III are clustered together closely. The last two growth phases cannot be separated due to the limited number of data points. However, even as they represent the changes of 24 h of fermentation, there is no high distance between the data points. This supports the findings that the stationary phase set in after 24 h and lasted until the end of the fermentation. The loading plot allocates the 35 different factors to the parts of the PCA and shows dependencies between the factors. While the growth phase I (substrate, 0, and 1 h) is dominated by most aldehydes, the carbohydrates fructose and glucose, and high pO_2_ and pH values, growth phase II (2–6 h) is connected to the later utilized carbohydrates ribose, saccharose, and mannose. Moreover, hexanol and ethyl hexanoate represent the later development of the second growth phase. Approaching the deceleration phase (growth phase III, 7–12 h), the high OD-value and 2-/3-methylbutanol are of importance, as are the sulfuric compounds DMDS and DMTS. The stationary phase (growth phase IV + V, 24–48 h) is dominated by the high concentration of the organic acids lactic and acetic acid, but also of the aroma compounds diacetyl, γ-nonalactone, and β-damascenone.

**Fig. 6:**
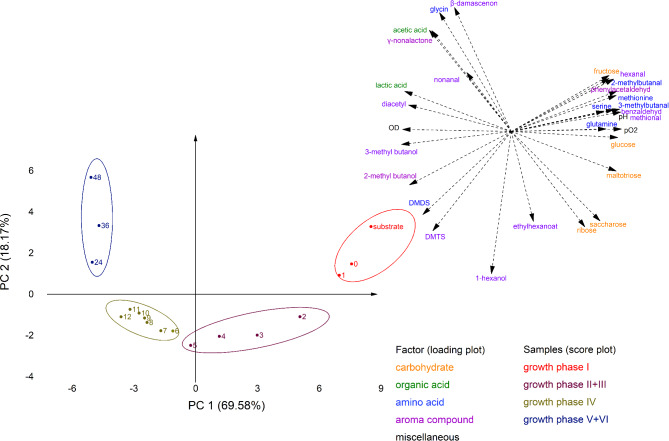
Principal Compound Analysis of all observed statistically significant changes in the fermentation of faba bean-based substrates with *Lactiplantibacillus plantarum* L879. The score plot (left) shows the samplings at different times in the fermentation. While the samples 2–5 h are distributed with a wide distance in between, the samples of growth phase III (6–12 h) are closely clustered together. The loading plot (right) describes the 30 influencing factors from the group’s carbohydrates, organic acids, amino acids, aroma compounds, and miscellaneous (like the pO_2_ value). To depict changes for all compounds despite their highly different concentration levels, the concentrations were normalized by dividing by the respective maximum values. PC 1 and PC 2 explain 87.8% of the total variation. (*n* = 4)

The changes in the faba bean-based substrate were comparable to the ones in lupines (Fig. 6). Several aldehydes with an unpleasant aroma connotation, the carbohydrates glucose and fructose, and high pH and pO_2_ values mark the first hours of the fermentation. With the ongoing fermentation (grow phase II, 2–5 h), profound changes in appearance and shifts in the aroma compounds, as well as in the sugar utilization, are perceivable. In the third growth phase (6–12 h), the pace of change is considerably slowed down, and the data points are closely clustered together. In contrast to lupines, more substantial changes have been perceivable in the last 24 h, and the data points are considerably separated along PC 2. However, as in lupines, the main factors are β-damascenone, diacetyl, γ-nonalactone, and the organic acids.

### Identification of suitable fermentation times using the space-time-yield

To identify the optimal duration of the fermentation, several aspects need to be considered. The maximum or minimum concentration of a compound can be a good indicator if that is really required for the product. Otherwise, the Space-Time-Yield (STY) is a more economic criterion, as it weighs the product yield against the fermentation time. Additionally, autolytic effects gain increasing importance once the LAB reach the stationary phase, and intracellular compounds might be released into the broth. Moreover, substrate consumption might be an additional factor that can be optimized, e.g., if a particular carbohydrate source needs to be exhausted (e.g., lactose in products that should be available for lactose intolerant consumers).

**Fig. 7:**
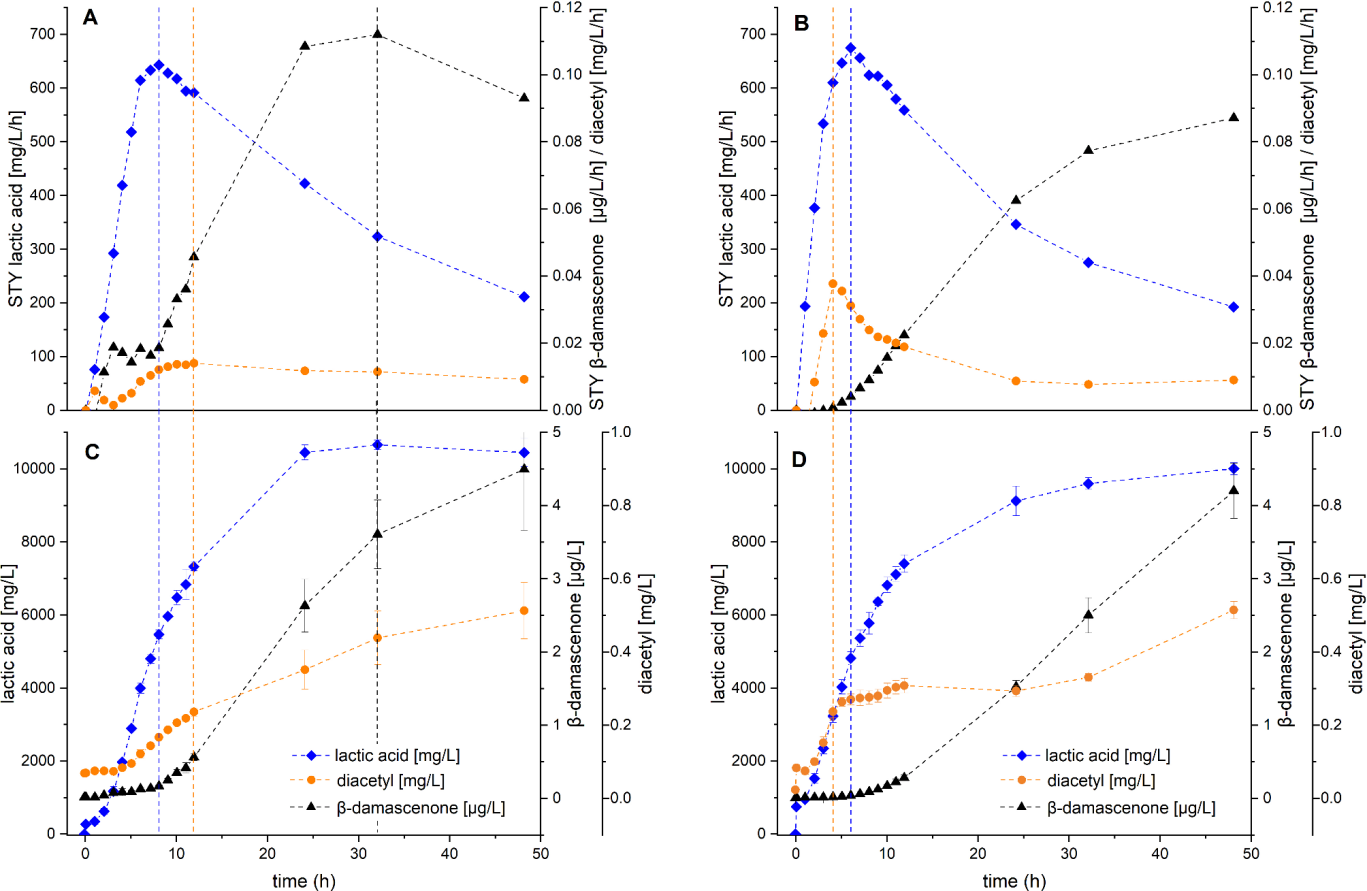
Space-Time-Yield and concentrations for lactic acid, diacetyl, and β-damascenone of *Lactiplantibacillus plantarum* L628 in lupine (A, C) and *Lactiplantibacillus plantarum* L879 in faba bean (B, C). Maximum values of STY are highlighted with vertical dotted lines, which facilitate the comparison of the STY with the concentration of the respective compound. The concentrations are the mean of four biological replicates

In this study, the negatively associated aldehydes were depleted within the first 3–9 h in both fermentations (compare Figs. 3 and 4). Sugar utilization is not a vital criterion as saccharose and maltose (only in faba bean) remain in the beverage after 48 h of fermentation and an entire consumption is not foreseeable. The STY was calculated for the sensorially positively associated compounds lactic acid, diacetyl, and β-damascenone and is depicted in Fig. 7 with the respective concentrations. The application of the STY for ethyl hexanoate and γ-nonalactone was deemed as misleading as those compounds were only present in concentrations inferior to their odor threshold. In L628 (lupine), the STY of lactic acid increased strongly until 8 h and declined afterward. For β-damascenone, the STY started to increase after approx. 8 h and reached its maximum after 32 h. The diacetyl concentration reached its maximum STY at 12 h, whereas the STY was almost at the same level between 10 and 12 h. In L879 (faba bean), the development of the lactic acid STY is comparable but reached its maximum already at 6 h. The slightly less steep increasing STY for β-damascenone can be observed in L879 as well. However, no maximum is perceivable, which might indicate that the optimum was not reached within 48 h. For diacetyl, the STY initially increased very strongly and declined exponentially after reaching its maximum at 4 h.

In both strains, the highest STY for lactic acid and diacetyl were within the first hours of growth, while β-damascenone was produced mainly in the latter phase of the fermentation. Especially as the concentration of β-damascenone strongly increased between 12 and 24 h, the fermentation should not be ended too early. Declining concentrations in the ongoing fermentations were observed neither for diacetyl nor for lactic acid. Therefore, fermentation times between 24 and 32 h can be recommended for both strains in the respective substrates.

## Conclusion

This study presented a statistically based approach to optimize the fermentation parameters temperature, inoculum cell concentration, and methionine addition for multiple strains in order to improve the fermentation of lupine- and faba bean-based substrates. Based on these experiments, a total of 48 models were identified to predict the impact of the fermentation parameters on the volatile and non-volatile metabolic products of the LAB. This enables to reduce the beany aroma and improve the sensory profile of a refreshing beverage.

Generally, the temperature was important for most models, whereas the inoculum cell concentration and the methionine addition were also impactful but to a lesser extent. Increasing fermentation temperatures led to reduced aldehyde and diacetyl concentrations and the acetic/lactic acid ratios, while the β-damascenone concentration increased strongly. Positively, the addition of methionine showed only a minor impact on the sulfuric compounds methional, DMS, DMDS, and DMTS. As this had no adverse impact on the sensory perception, methionine addition might help to improve the protein bioavailability without severe adverse effects on the overall aroma of the final beverage.

To further support the reliability of the prediction models, the validation was performed at independent parameter values within the original design space. Thereby, 39 of the 48 models were confirmed to be statistically reliable, and their outcome was shown to be reproducible.

In the bioreactor, LAB grew well on both legume-based substrates, highly decreased the dissolved oxygen, and reached pH values of ≤ 4.0 in less than 6.5 h. While glucose and fructose were primarily metabolized, the ribose concentration increased until fructose was depleted and decreased afterward. In lupines, this diauxie was also perceivable at the optical density. Saccharose, mannose (lupine only), and maltose (faba bean only) were degraded later in the fermentation and to a lesser degree. The substantial oxygen utilization, in combination with considerable acetic acid production, clearly shows the impact of oxygen on homofermentative LAB fermentations. In the aroma-active compounds, the concentrations of most aldehydes decreased fast and strongly within the first hours of fermentation. In contrast, the peasant compounds diacetyl, γ-nonalactone, and β-damascenone increased mainly in the latter fermentation time. This proves that LAB fermentation is a beneficial process for the fast removal of aldehydes with an unpleasant aroma impression. However, it was also shown that the fermentation should not be terminated prematurely, as positive aroma compounds might be produced to a high extent later in the fermentation.

Finally, recommendations for the LAB fermentation in the production of lupine- and faba bean-based beverages were presented by comparing the Space-Time-Yield and the absolute concentrations of the most relevant changing compounds (see Table 5).

**Table 5:** Recommended fermentation parameters for the production of sensorily appealing lupine- and faba bean-based beverages

	model parameter			
legume	fermentation temperature	inoculum size	methionine addition	fermentation time
lupine	38 °C	73∙10^6^ cells/mL	8.4 mg/L	24–32 h
faba bean	39 °C	96∙10^6^ cells/mL	11.4 mg/L	24–32 h

Based on the presented findings, further research should substantiate the effect of a methionine addition on the protein bioavailability in legume-based beverages. This should be combined with a close focus on sensory perception to further confirm that no adverse effects appear due to the methionine addition. Moreover, further efforts should elucidate in comparative fermentations if the exclusion of oxygen leads to a significant change in the metabolite production or whether other electron acceptors in the complex media would substitute oxygen. This might answer whether the acetic acid production might be modulated by introducing or excluding specific electron acceptors in the fermentation.

## Electronic supplementary material

Below is the link to the electronic supplementary material.

Supplementary Material 1

Supplementary Material 2

## Data Availability

The authors declare that the data supporting the findings of this study are partially available within the paper and its Supplementary Information files. Should any further data files be needed they are available from the corresponding author upon request.
