# Supplementary material for: Optimizing the fermentation parameters in the Lactic Acid Fermentation of Legume-based Beverages– a statistically based fermentation
Source: Microb Cell Fact. 2024 Sep 19;23:253. doi: 10.1186/s12934-024-02522-x (PMC11411822; doi:10.1186/s12934-024-02522-x)
Supplement: Supplementary file 1 — Supplementary Material 1 [file 12934_2024_2522_MOESM1_ESM.docx]

Table S1: Identification of LAB strains by MALDI-TOF and 16S Sequencing

| strain | Identification MALDI | Score value | 16S Sequencing | Seq | % |
| --- | --- | --- | --- | --- | --- |
| L758 | *Lactiplantibacillus plantarum* | 2.28 | *Lactiplantibacillus plantarum/argentoratensis* | 16S | 99.02 |
| L628 | *Lactiplantibacillus plantarum* | 2.44 | *Lactiplantibacillus plantarum/pentosus/argentoratensis* | 16S | 99.82 |
| L1264 | *Lactiplantibacillus rhamnosus* | 2.06 | *Lacticaseibacillus rhamnosus* | 16S | 99.34 |
| L762 | *Lactiplantibacillus plantarum* | 2.44 | *Lactiplantibacillus plantarum/pentosus/argentoratensis* | 16S | 100 |
| L879 | *Lactiplantibacillus plantarum* | 1.76 | *Lactiplantibacillus plantarum/pentosus/argentoratensis* | 16S | 99.86 |
| L1276 | *Lactiplantibacillus plantarum* | 2.32 | *Lactiplantibacillus plantarum* | 16S | 99.54 |

Table S2: Validation data for the sugar analysis (HPAEC-PAD)

|  | glycerol | erythritol | xylitol | sorbitol | mannitol | arabinose | mannose | glucose |
| --- | --- | --- | --- | --- | --- | --- | --- | --- |
| linear range [mg/L] | 4–190 | 3.5–157 | 3–137 | 3–135 | 3–132 | 6–283 | 6–268 | 6–263 |
| R² | 0.9985 | 0.9998 | 0.9999 | 0.9998 | 0.9998 | 0.9956 | 0.9997 | 0.9995 |
| recovery (lupine) | 96.4% | 105.9% | 97.7% | 103.9% | 97.1% | 97.0% | 94.5% | 102.8% |
| recovery (faba bean) | 101.9% | 102.9% | 103.1% | 107.0% | 91.1% | 98.9% | N/A^1^ | 105.7% |
| LOD [mg/L] | 0.11 | 0.05 | 0.07 | 0.09 | 0.09 | 0.17 | 0.11 | 0.12 |
| LOQ [mg/L] | 0.33 | 0.14 | 0.22 | 0.28 | 0.26 | 0.51 | 0.32 | 0.36 |
|  |  |  |  |  |  |  |  |  |
|  | xylose | fructose | ribose | saccharose | maltulose | maltose | maltotriose |  |
| linear range [mg/L] | 6–283 | 6–257 | 6–268 | 6–285 | 6–275 | 56–2540 | 54–2450 |  |
| R² | 0.9999 | 0.9992 | 0.9992 | 0.9997 | 0.9996 | 0.9985 | 0.9998 |  |
| recovery (lupine) | 97.8% | 93.2% | 96.9% | 97.5% | 114.0% | 106.5% | 83.5% |  |
| recovery (faba bean) | 105.2% | 100.7% | 102.0% | 94.7% | 115.3% | 101.7% | 91.0% |  |
| LOD [mg/L] | 0.20 | 0.34 | 0.26 | 0.75 | 0.82 | 0.55 | 1.57 |  |
| LOQ [mg/L] | 0.59 | 1.03 | 0.76 | 2.28 | 2.49 | 1.67 | 4.76 |  |
|  |  |  |  |  |  |  |  |  |
| ^1^As no mannose peak was found in the faba bean samples, no recovery analysis was performed | | | | | | |  |  |

Table S3: Validation data for the organic acid analysis (HPLC-UV)

|  | lactic acid | acetic acid |
| --- | --- | --- |
| linear range [mg/L] | 2.25–450 | 4–800 |
| R² | 0.99974 | 0.99973 |
| recovery (lupine) | 103% | 100% |
| recovery (faba bean) | 101% | 98% |
| LOD [mg/L] | 0.006 | 0.004 |
| LOQ [mg/L] | 0.020 | 0.013 |

Table S4: Validation data for the amino acid analysis (LC-MS)

|  | Valin | Alanin | Arginin | Asparagin | Glutamin | Glutaminsäure | Glycin | Isoleucin |
| --- | --- | --- | --- | --- | --- | --- | --- | --- |
| linear range [mg/L] | 0.235–11.8 | 0.193–9.65 | 0.436–21.8 | 0.280–14.0 | 0.297–14.9 | 0.300–15.0 | 0.161–8.05 | 0.275–13.8 |
| r | 0.99948 | 0.99966 | 0.99911 | 0.99955 | 0.99959 | 0.9989 | 0.99927 | 0.99854 |
| R² | 0.999 | 0.999 | 0.998 | 0.999 | 0.999 | 0.998 | 0.999 | 0.997 |
| LOD [mg/L] | 0.0019 | 0.0048 | 0.0030 | 0.0020 | 0.0072 | 0.0033 | 0.0082 | 0.0049 |
| LOQ [mg/L] | 0.0062 | 0.0159 | 0.0100 | 0.0067 | 0.0241 | 0.0110 | 0.0273 | 0.0162 |
|  |  |  |  |  |  |  |  |  |
|  | Serin | Threonin | Tryptophan | Tyrosin | Asparaginsäure | Leucin | Phenylalanin | Histidin |
| linear range [mg/L] | 0.214–10.7 | 0.246–12.3 | 0.410–20.5 | 0.372–18.6 | 0.324–16.2 | 0.275–13.8 | 0.352–17.6 | 0.335–16.8 |
| r | 0.99951 | 0.99946 | 0.99931 | 0.99967 | 0.99915 | 0.99793 | 0.99882 | 0.99923 |
| R² | 0.999 | 0.999 | 0.999 | 0.999 | 0.998 | 0.996 | 0.998 | 0.998 |
| LOD [mg/L] | 0.0026 | 0.0029 | 0.0025 | 0.0026 | 0.0145 | 0.0141 | 0.0024 | 0.0050 |
| LOQ [mg/L] | 0.0087 | 0.0096 | 0.0082 | 0.0087 | 0.0483 | 0.0471 | 0.0080 | 0.0167 |
|  |  |  |  |  |  |  |  |  |
|  | Methionin | Prolin | Lysin |  |  |  |  |  |
| linear range [mg/L] | 0.291–14.6 | 0.245–12.3 | 0.296–14.8 |  |  |  |  |  |
| r | 0.99959 | 0.99557 | 0.9988 |  |  |  |  |  |
| R² | 0.999 | 0.991 | 0.998 |  |  |  |  |  |
| LOD [mg/L] | 0.0022 | 0.0022 | 0.0067 |  |  |  |  |  |
| LOQ [mg/L] | 0.0074 | 0.0074 | 0.0223 |  |  |  |  |  |

Table S5: Validation data for the aroma analysis (GC-MS)

|  |  | **lupine** | | | | **faba bean** | | | |
| --- | --- | --- | --- | --- | --- | --- | --- | --- | --- |
| **aroma compound** | **quantifier ions (m/z)** | **Calibration Function [-]^1^** | **R²** | **LOD [µg/L]** | **LOQ [µg/L]** | **Calibration Function [-]^1^** | **R²** | **LOD [µg/L]** | **LOQ [µg/L]** |
| dimethyl sulfide | 61 | 0.00688 | 0.987 | 0.124 | 0.413 | 0.00703 | 0.990 | 0.148 | 0.492 |
| 3-methyl butanal | 58 | 0.02136 | 0.994 | 0.254 | 0.846 | 0.03067 | 0.990 | 0.142 | 0.472 |
| 2-methyl butanal | 57 | 0.54818 | 0.994 | 0.017 | 0.057 | 0.27355 | 0.986 | 0.024 | 0.080 |
| dimethyl disulfide | 94 | 0.22897 | 0.997 | 0.003 | 0.009 | 0.24399 | 0.991 | 0.003 | 0.010 |
| hexanal | 56 | 0.40741 | 0.981 | 0.014 | 0.045 | 0.27189 | 0.984 | 0.019 | 0.062 |
| methional | 48 | 0.00649 | 0.984 | 0.305 | 1.017 | 0.00604 | 0.988 | 0.356 | 1.185 |
| benzaldehyde | 77 | 1.07301 | 0.994 | 0.008 | 0.026 | 1.32397 | 0.998 | 0.006 | 0.020 |
| dimethyl trisulfide | 126 | 1.48218 | 1.000 | 0.001 | 0.002 | 1.79094 | 0.998 | 0.001 | 0.002 |
| ethylhexanoate | 88 | 2.18081 | 0.995 | 0.001 | 0.002 | 2.37357 | 0.996 | 0.001 | 0.003 |
| phenylacetaldehyde | 91 | 0.19316 | 0.998 | 0.025 | 0.083 | 0.20716 | 0.996 | 0.030 | 0.101 |
| nonanal | 57 | 6.24062 | 0.988 | 0.002 | 0.005 | 9.84737 | 0.998 | 0.002 | 0.008 |
| (*E*)-2-nonenal | 43 | 2.52082 | 0.983 | 0.006 | 0.018 | 3.61539 | 0.998 | 0.007 | 0.023 |
| (*E,E*)-2,4-nonadienal | 81 | 10.96011 | 0.987 | 0.002 | 0.005 | 17.45055 | 0.998 | 0.002 | 0.007 |
| gamma-nonalactone | 85 | 0.27178 | 0.993 | 0.014 | 0.047 | 0.27546 | 0.993 | 0.016 | 0.053 |
| beta-damascenone | 69 | 4.10094 | 0.982 | 0.001 | 0.004 | 4.41146 | 0.991 | 0.001 | 0.004 |
| 3-methyl butanol | 55 | 0.00017 | 0.981 | 0.536 | 1.788 | 0.00046 | 0.981 | 0.345 | 1.151 |
| 2-methyl butanol | 56 | 0.00021 | 0.977 | 1.463 | 4.876 | 0.00043 | 0.986 | 0.667 | 2.224 |
| 1-hexanol | 56 | 0.00130 | 0.986 | 0.048 | 0.160 | 0.00329 | 0.990 | 0.020 | 0.067 |
|  |  |  |  |  |  |  |  |  |  |
| ^1^The calibration function is the slope of the calibration curve with the concentration ratio c(analyte)/c(internal standard) on the abscissa and the area ratio A(analyte)/A(internal standard) on the ordinate | | | | | | | | | |

Table S6: Identification of LAB strains by MALDI-TOF in the bioreactor experiments

| Experiment | Substrate | Identification MALDI | Score value |
| --- | --- | --- | --- |
| L628 - 1 | lupine | *Lactiplantibacillus plantarum* | 2.35 |
| L628 - 2 | lupine | *Lactiplantibacillus plantarum* | 2.36 |
| L628 - 3 | lupine | *Lactiplantibacillus plantarum* | 2.36 |
| L628 - 4 | lupine | *Lactiplantibacillus plantarum* | 2.35 |
| L879 - 1 | faba bean | *Lactiplantibacillus plantarum* | 2.10 |
| L879 - 2 | faba bean | *Lactiplantibacillus plantarum* | 2.13 |
| L879 - 3 | faba bean | *Lactiplantibacillus plantarum* | 2.14 |
| L879 - 4 | faba bean | *Lactiplantibacillus plantarum* | 2.06 |
